# Up‐regulation of cofilin‐1 in cell senescence associates with morphological change and p27^kip1^‐mediated growth delay

**DOI:** 10.1111/acel.13288

**Published:** 2020-12-18

**Authors:** Cheng‐Han Tsai, Chun‐Yuan Chang, Bing‐Ze Lin, Yu‐Lou Wu, Meng‐Hsiu Wu, Liang‐Tin Lin, Wen‐Chien Huang, Jonathan D. Holz, Tzong‐Jen Sheu, Jhih‐Shian Lee, Richard N. Kitsis, Pei‐Han Tai, Yi‐Jang Lee

**Affiliations:** ^1^ Department of Biomedical Imaging and Radiological Sciences National Yang‐Ming University Taipei Taiwan; ^2^ Department of Surgery Division of Thoracic Surgery MacKay Memorial Hospital Taipei Taiwan; ^3^ Department of Biology University of Rochester Rochester NY 14642 USA; ^4^ Department of Orthopaedics Center for Musculoskeletal Research University of Rochester School of Medicine Rochester NY 14642 USA; ^5^ Departments of Medicine (Cardiology) and Cell Biology and Wilf Family Cardiovascular Research Institute Albert Einstein College of Medicine Bronx, New York NY USA; ^6^ Graduate Institute of Oral Biology School of Dentistry National Taiwan University Taipei Taiwan; ^7^ Cancer Progression Research Center National Yang‐Ming University Taipei 11221 Taiwan

**Keywords:** cofilin‐1, growth arrest, morphology, p27^Kip1^, senescence, TEAD1

## Abstract

Morphological change is an explicit characteristic of cell senescence, but the underlying mechanisms remains to be addressed. Here, we demonstrated, after a survey of various actin‐binding proteins, that the post‐translational up‐regulation of cofilin‐1 was essential for the reduced rate of actin depolymerization morphological enlargement in senescent cells. Additionally, up‐regulated cofilin‐1 mainly existed in the serine‐3 phosphorylated form, according to the 2D gel immunoblotting assay. The up‐regulation of cofilin‐1 was also detected in aged mammalian tissues. The over‐expression of wild‐type cofilin‐1 and constitutively phosphorylated cofilin‐1 promoted cell senescence with an increased cell size. Additionally, senescent phenotypes were also reduced by knockdown of total cofilin‐1, which led to a decrease in phosphorylated cofilin‐1. The senescence induced by the over‐expression of cofilin‐1 was dependent on p27^Kip1^, but not on the p53 and p16^INK4^ expressions. The knockdown of p27^Kip1^ alleviated cell senescence induced by oxidative stress or replicative stress. We also found that the over‐expression of cofilin‐1 induced the expression of p27^Kip1^ through transcriptional suppression of the transcriptional enhancer factors domain 1 (TEAD1) transcription factor. The TEAD1 transcription factor played a transrepressive role in the p27^Kip1^ gene promoter, as determined by the promoter deletion reporter gene assay. Interestingly, the down‐regulation of TEAD1 was accompanied by the up‐regulation of cofilin‐1 in senescence. The knockdown and restoration of TEAD1 in young cells and old cells could induce and inhibit p27^Kip1^ and senescent phenotypes, respectively. Taken together, the current data suggest that cofilin‐1/TEAD1/p27^Kip1^ signaling is involved in senescence‐related morphological change and growth arrest.

## Graphical abstract

Triggers of senescence, such as replicative stress or oxidative stress, can induce the expression of cofilin‐1 to influence of cell morphology. Up‐regulated cofilin‐1 would enhance the expression of p27kip1 gene by suppressing the TEAD‐1 that transrepresses p27kip1 and lead to senescence‐associated growth delay.

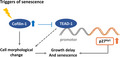

## 1 INTRODUCTION

Cellular senescence is a state of irreversible growth arrest that prevents the indefinite proliferation of mammalian cells. Replicative senescence usually refers to a reduced proliferative rate, shortening of telomeres, and morphological enlargement (Young, 2018). The hallmarks of senescence have been expanded to include alterations to signaling pathways (Hernandez‐Segura et al., 2018). Among them, morphological alterations occur in response to these signaling pathways and exhibit an enlarged cell size and flattened shape, increased lysosomal content, and nuclear changes such as a loss of the lamin B1 and lamin B receptors. Morphological alteration during cell senescence is believed to be related to the re‐organization of the actin cytoskeleton (Biran et al., 2015). However, the underlying mechanisms still need to be addressed.

The actin depolymerizing factor (ADF)/cofilin family encodes ~19 kD actin‐binding proteins in mammals and includes cofilin‐1, cofilin‐2, and ADF (Bernstein & Bamburg, 2010). Cofilin‐1 and ADF are co‐expressed in non‐muscle cells, although cofilin‐1 is usually predominantly expressed in various cell types (Hotulainen et al., 2005). Moreover, cofilin‐1 activity is regulated by the Rho/ROCK/LIM kinase signaling pathway. This pathway phosphorylates the serine‐3 residue of cofilin‐1 and thereby weakens the activity of cofilin to sever actin filaments (Elam et al., 2017). Interestingly, several lines of evidence have indicated that the cytoplasmic rods formed from ADF/cofilin and actin in a 1:1 stoichiometry are increased in the brains of patients suffering from Alzheimer's disease, a neurodegenerative disorder usually found in mid‐ to late‐age populations (Alsegiani & Shah, 2020; Bamburg & Bernstein, 2016). Because cofilin‐1 is ubiquitously expressed in all mammalian cells, investigation into whether cofilin‐1 is also involved in cell senescence within different organs is of interest.

G0/G1 phase arrest is one of the important characteristics of senescence. The INK4 and CIP/KIP protein families are primarily cyclin‐dependent kinase inhibitors (CKIs) that inhibit G0/G1 phase progression (Reynisdottir et al., 1995). The p53/p21^Cip1^ and retinoblastoma (Rb)/p16^INK4^ signaling pathways are well‐established mechanisms that link cell cycle arrest and senescence (McHugh & Gil, 2018; Ohtani et al., 2004). The cell cycle regulator p27^Kip1^ is reported to be associated with senescence through the PTEN‐Skp2 signaling pathway (Lin et al., 2010). What is more, the p27^Kip1^ regulation of cell cycle progression and oncogenic signaling can also be controlled by cell shape and mechanical/cytoskeletal tension (Huang et al., 1998; Jang et al., 2017). The enforced expression of cofilin‐1 has been shown to cause actin cytoskeletal destabilization and G1 phase arrest via the induction of p27^Kip1^ (Tsai et al., 2009). The ability of morphological change to activate specific cell cycle regulatory pathways and thereby promote senescent phenotypes has not been fully studied yet.

The transcriptional enhancer factors domain (TEAD) protein family contains four isoforms (TEAD1/2/3/4), which are highly conserved in mammalian cells and are related to gene transcription during development and oncogenic activation (Zhou et al., 2016). TEAD proteins share a common TEA DNA binding domain, but their transcriptional activities are determined by bound co‐activators (Hori et al., 2020). The interaction between TEADs and the YAP/TAZ transcriptional coactivator is known to counteract the Hippo tumor suppressive pathway (Thompson, 2020). Additionally, TEAD is important for mediating Hippo signaling in cell proliferation, contact inhibition, and control of organ size (Ota & Sasaki, 2008). TEADs have also been reported to be required for YAP deficiency‐induced senescence (‘Correction: YAP/TEAD‐Mediated Transcription Controls Cellular Senescence’, 2017). How TEAD transcription factors regulate cell senescence remains to be addressed.

In this study, we found that cofilin‐1 was up‐regulated in cells exhibiting senescence‐related phenotypes, including an increase of stress fibers and morphological change. Interestingly, the serine‐3 phosphorylated form of cofilin‐1 was also increased. Upon over‐expression, both wild‐type cofilin‐1 and phosphomimetic mutant cofilin‐1 induced similar levels of SA‐β‐gal activity and cell enlargement. The manipulation of cofilin‐1 can ablate cell senescence through the regulation of p27^Kip1^, but not p53 or p16^INK4^. A survey of p27^Kip1^ gene promoters revealed that a TEAD1 transcription factor had a transrepressive effect on p27^Kip1^ gene expression. TEAD1 was down‐regulated during cell senescence and by the over‐expression of cofilin‐1. These data provide evidence that the cofilin‐1/TEAD1/p27^Kip1^ signaling axis represents a novel mechanism for the regulation of cell senescence.

## 2 RESULTS

### 2.1 The morphological change of senescent cells is associated with an altered actin polymerization/depolymerisation rate and cofilin‐1 level

**Figure 1:**
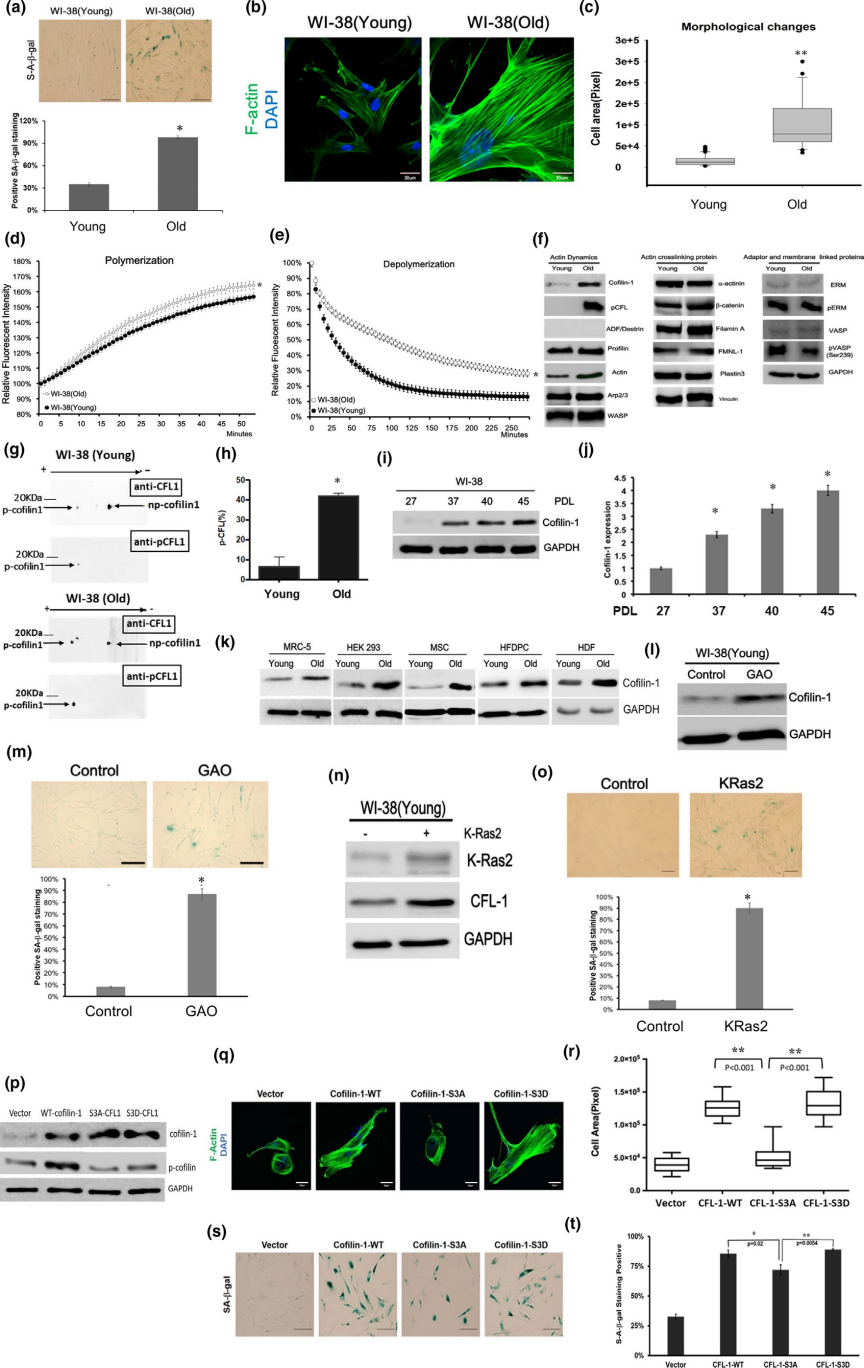
Up‐regulation of cofilin‐1 (CFL‐1) in cell senescence. (a) SA‐β‐gal staining for young cells and old cells. The percentage of SA‐β‐gal expressing cells out of the total cells was performed to compare it between young cells and old cells. (b) Staining of actin filaments using fluorescein‐conjugated phalloidin. (c) Measurement of cell areas using the cell morphology analyzer. *N* = 50 and 28 for young cells and senescent cells, respectively. (d, e) Comparison of the actin polymerization rate and depolymerization rate between young cells and old cells using the pyrene‐actin polymerization assay. The error bars are the standard deviation obtained from triplicate experiments. (f) Survey and comparison of actin‐associated proteins in young cells and old cells using Western blot analysis. Arp2/3, actin‐related protein 2/3; WASP, Wiskott–Aldrich syndrome protein; FMNL1, Formin‐like protein 1; ERM, ezrin, radixin, and moesin; VASP, vasodilator‐stimulated phosphoprotein. GAPDH, glyceraldehyde‐3‐phosphate dehydrogenase. (g) The 2D gel blotting assay for analyzing the level change of phosphorylated cofilin‐1 (p‐cofilin‐1) and non‐phosphorylated cofilin‐1 (np‐cofilin‐1) between young cells and old cells. (H) Increase of p‐cofilin‐1 ratios related to the total cofilin‐1 by quantification of 2D gel blotting. (i) Detection of the cofilin‐1 protein expression in different population doubling levels (PDLs) of WI‐38 cells. (j) Densitometric quantification of cofilin‐1 in different PDLs of WI‐38 cells. (k) Western blot analysis of cofilin‐1 expression in different cell types at young stages (<3 passages) and old stages (>10 passages). (l) Galactose oxidase (GAO) (1 unit/ml) induced cofilin‐1 expression in young WI‐38 cells. Treatment time: 24 h. (m) GAO promotes the expression of SA‐β‐gal in treated cells. (n, o) Over‐expression of K‐Ras2 induced cofilin‐1 expression and SA‐β‐gal activity in WI‐38 cells, respectively. (p) Transduction of wild‐type, S3A mutant, and S3D mutant cofilin‐1 into young WI‐38 cells, separately. (q) The fluorescein‐conjugated phalloidin staining of the actin cytoskeleton. (r) Comparison of cell areas between cells transduced with different forms of cofilin‐1 (*N* = 15 for each group). (s) Visualization of SA‐β‐gal staining. (t) Percentages of positive SA‐β‐gal stained cells (*N* = 100 for each group). Scale bar: 100 μm for bright field images and 30 μm for fluorescent images *: *p* < 0.05, **: *p* < 0.01

Compared with young WI‐38 lung fibroblasts (lower population doubling level (PDL) <27), senescent cells (PDL > 37) exhibited an enhanced SA‐β‐gal level, as report before (Figure 1a; (Itahana et al., 2013)). Additionally, senescent cells exhibited a significant G1 phase arrest; reduced Ki‐67 expression; delayed growth rate; up‐regulated p53, p16^INK4^; p21^Cip1^, p27^Kip1^, and γH2AX; and shorter telomere length compared with young cells (Figure S1). Given these senescence‐associated changes, we compared the actin organization and morphological differences between young and senescent cells. Images of senescent cells stained with fluorescein‐conjugated phalloidin showed a greater number of stress fibers and were larger in size than the young cells (Figure 1b). The cell size of fluorescein‐conjugated phalloidin stained WI‐38 cells was then quantified using a cell morphology analyzer (Figure S2). The areas of senescent cells were significantly larger than that of the young cells (Figure 1c). We also compared the lung tissue sections resected from young mice (6 weeks old) and old mice (80 weeks old) by staining with fluorescein‐conjugated phalloidin. This revealed that the cell shape of old lung tissue was more irregular or larger than that of the young lung tissue (Figure S3a). The senescent markers, including SA‐β‐gal and p53, were also mainly detected in the cells with irregular shapes (Figure S3b). Additionally, the radial alveolar counts (RAC) and measurement of the alveolar areas were used to quantify the morphological difference of lung tissues from young and old mice (Betz et al., 1997). The RAC and the alveolar areas of the old lung tissue sections were lower and larger than that of the young tissue sections, respectively (Figure S3c and d). Additionally, the actin polymerization and depolymerization rates were compared between young cells and senescent cells using a pyrene‐conjugated actin polymerization assay. The results showed that the cell lysates obtained from the senescent cells exhibited a higher actin polymerization rate than the lysates from the young cells (Figure 1d). Moreover, the actin depolymerization rate of the senescent cell was significantly slower than that of the young cells using the same cell lysates (Figure 1e). Subsequently, we compared 17 actin‐associated proteins involved in actin polymerization/depolymerization, cytoskeletal crosslinking, and actin‐extracellular matrix interaction using Western blot analysis. Interestingly, of the 17 proteins, cofilin‐1 was the main actin‐associated protein up‐regulated in the senescent cells (Figure 1f and Figure S4). It was found that a serine‐3 phosphorylated form of cofilin‐1 and total cofilin‐1 were both up‐regulated, so a two‐dimensional (2D) gel immunoblotting assay was used to examine the changes in cofilin‐1, with or without phosphorylation. The data show that phosphorylated cofilin‐1 (p‐cofilin‐1) was significantly increased in senescent cells compared with non‐phosphorylated cofilin‐1 (np‐cofilin‐1; Figure 1g). The use of densitometry showed that the ratios of p‐cofilin‐1 to total cofilin‐1 in young cells and old cells were approximately 10% and 40%, respectively (Figure 1h). On the contrary, ADF/destrin was not expressed in these cells. Furthermore, the up‐regulation of the cofilin‐1 protein was accompanied by an increase of PDLs (Figure 1i and j). Besides WI‐38 cells, the up‐regulation of cofilin‐1 was also detected in several human cell lines exhibiting senescent phenotypes after serial passages, including MRC‐5 lung fibroblasts, human mesenchymal stem cells (hMSC (Hung et al., 2002), hair follicle dermal papilla cells (HFDPC), and dermal fibroblasts (Figure 1k and Figure S5). In addition to replicative senescence, we also examined whether cofilin‐1 would be up‐regulated by oxidative stress‐induced premature senescence. Galactose oxidase (GAO), an enzyme that can catalyze D‐galactose to D‐galactohexodialdose and generate endogenous H_2_O_2_ in the presence of oxygen, was used to treat the WI‐38 cells (Wang et al., 1998). The results showed that young WI‐38 cells treated with GAO exhibited up‐regulated cofilin‐1 (Figure 1l) and increased SA‐β‐gal levels (Figure 1m). The exposure of cells to an H_2_O_2_ solution also induced cofilin‐1 and increased the SA‐β‐gal level (Figure S6). Moreover, transduction of the K‐Ras2 oncogene into cells concomitantly induced cofilin‐1 (Figure 1n) and increased the SA‐β‐gal level (Figure 1o). These data indicate that the up‐regulation of cofilin‐1 occurs in cell senescence induced by various growth stresses.

Next, we asked whether the over‐expression of wild‐type cofilin‐1 and constitutively phosphorylated mutant cofilin‐1 (S3D) would similarly promote morphological change and senescence in young cells, as both the total and phosphorylated cofilin‐1 were up‐regulated in senescent cells. The transduction of wild‐type, S3D, and S3A (constitutively non‐phosphorylated form) mutant cofilin‐1 was confirmed by detecting the levels of the total cofilin‐1 and serine‐3 phosphorylated cofilin‐1 (Figure 1p). Compared with the cells transduced with S3A cofilin‐1, both wild‐type cofilin‐1 and S3D cofilin‐1 transduced cells showed an increased formation of stress fibers and increased cell areas (Figure 1q and r). The percentage of SA‐β‐gal staining was increased in the cells transduced with different forms of cofilin‐1, although wild‐type cofilin‐1 and S3D cofilin‐1 exhibited about 10% more positive stained cells than S3A cofilin‐1 (Figure 1s and t). The growth rates of the cells transduced with different forms of cofilin‐1, however, did not show significant differences (Figure S7), suggesting that both phosphorylated and non‐phosphorylated cofilin‐1 would induce senescent‐associated growth delays. As phosphorylated cofilin‐1 is less effective in binding to actin filaments, we also investigated whether the direct depletion of the actin‐binding activity of cofilin‐1 would influence cell senescence. An actin‐binding defective mutant form of cofilin‐1, named K112Q/K114Q, was created, as reported before (Moriyama et al., 1992). This mutant form of cofilin‐1 was transduced to normal WI‐38 cells for the detection of the actin cytoskeletal organization and SA‐β‐gal activity. Compared with wild‐type cofilin‐1 and S3D cofilin‐1, the ectopic expression of K112Q/K114Q mutant cofilin‐1 also increased the actin cytoskeleton, cell areas, and SA‐β‐gal staining (Figure S8). Hence, phosphorylated cofilin‐1 should contribute to an increase of stress fibers and cell size in senescent cells compared with non‐phosphorylated cofilin‐1, even though both forms affect cell growth.

### 2.2 Up‐regulation of cofilin‐1 in aged mammalian tissues

**Figure 2:**
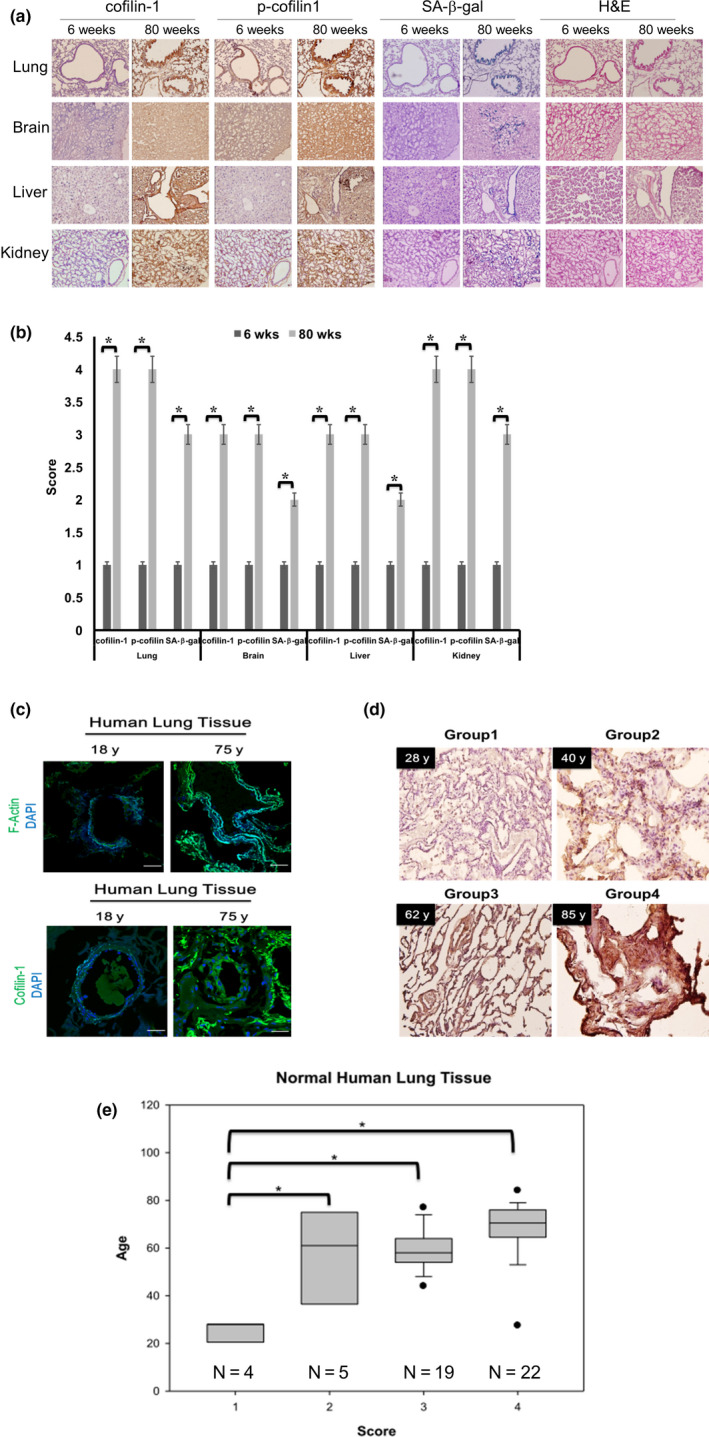
Up‐regulation of the total and phosphorylated cofilin‐1 in aged mouse tissues and aged human tissues. (a) IHC staining of the cofilin‐1 and p‐cofilin‐1 expressions, and SA‐β‐gal staining in consecutive cryosections (5 μm) of tissues, including lung, brain, liver, and kidney, from 6‐ and 80‐week‐old mice. Tissues were also stained by H&E. Scale bar: 100 μm. (b) Scoring of IHC results and SA‐β‐gal staining. (c) Fluorescent staining of actin cytoskeleton and cofilin‐1 in human lung tissue sections of donors at 18 years old and 75 years old. Scale bar: 100 μm. (d) IHC staining of cofilin‐1 in cryosections of normal human lung tissues from donors at different ages. (e) Results were classified into four groups according to the IHC scores of cofilin‐1 from 1 to 4. N represents the donor numbers belonging to different intensity and percentage distributions of IHC staining. *: *p* < 0.05

Next, we examined the levels of cofilin‐1 in different tissues of aged and young mice. The consecutive tissue cryosections obtained from the lungs, brain, liver, and kidneys of 6‐week‐old and 80‐week‐old mice were subjected to immunohistochemical (IHC) staining of cofilin‐1 and phosphorylated cofilin‐1, as well as tissue SA‐β‐gal staining. The results showed that the up‐regulation of cofilin‐1 and p‐cofilin‐1 was accompanied by an increased SA‐β‐gal activity in aged tissue sections compared with young tissue sections (Figure 2a). The IHC and SA‐β‐gal staining of these tissue sections were quantified by scoring, and the findings between young and old tissues were compared (Figure 2b). We also examined the human lung tissue sections of young (18 years old) and aged (75 years old) donors, and the latter expressed higher cofilin‐1 levels and increased phalloidin staining compared with the former (Figure 2c). Additionally, arbitrary scores were assigned for the IHC analysis of lung tissues from a small cohort of 50 donors at different ages (Figure 2d). The cohort study showed that higher scores (>2) were detected in individuals over 40 years old (Figure 2e). Therefore, cofilin‐1 up‐regulation is not only a characteristic of cultured senescent cells, but also aged mammalian tissues.

### 2.3 Increase of protein stability contributes to cofilin‐1 up‐regulation in cell senescence

**Figure 3:**
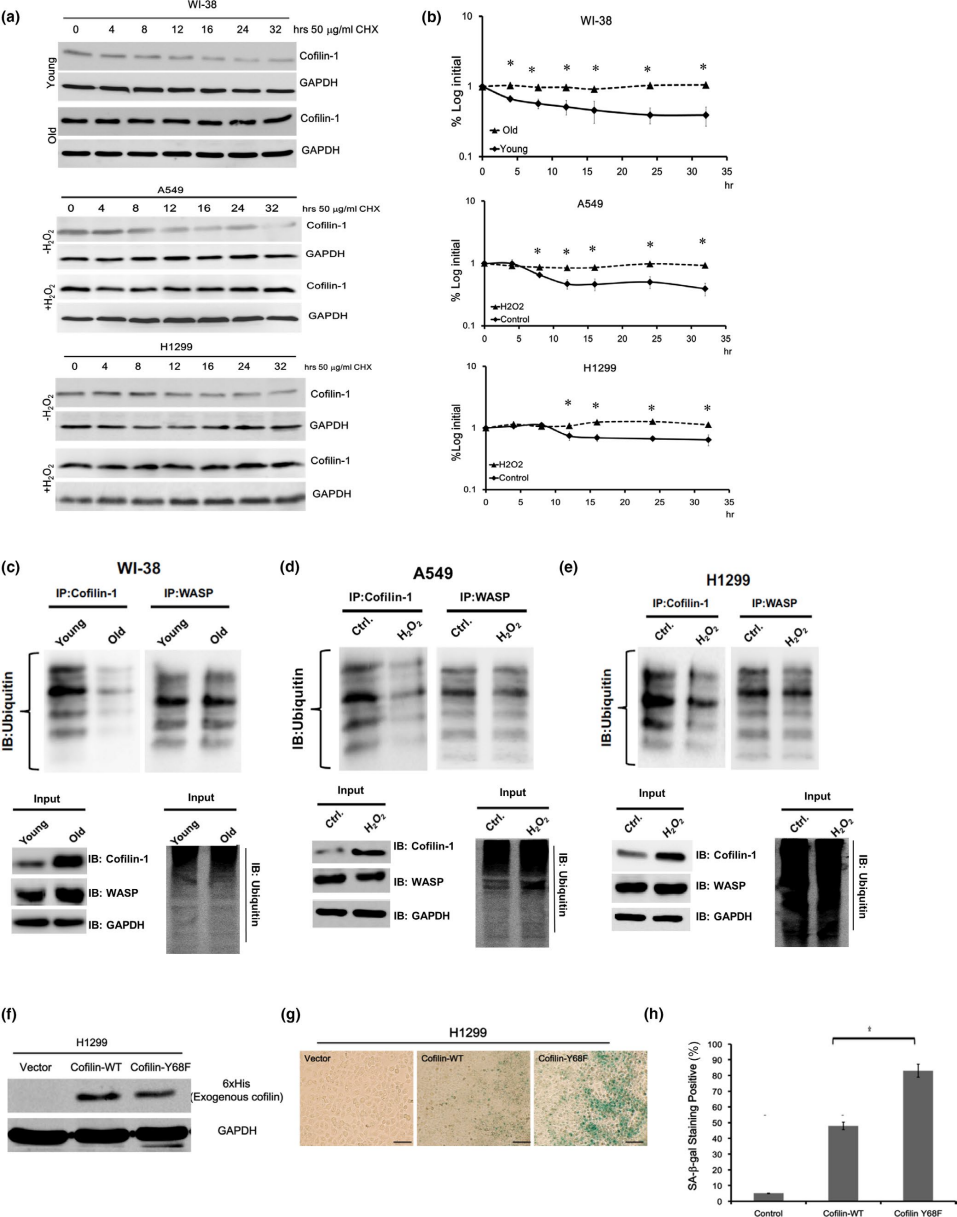
Decrease of cofilin‐1 protein ubiquitination in cell senescence. (a) Western blot analysis for the detection of the cofilin‐1 stability in young cells and old cells using 50 μg/ml of cycloheximide (CHX) to block the protein translation. Old WI‐38 cells were obtained by replicative senescence, and old A549 cells and H1299 cells were obtained by H_2_O_2_ treatment. (b) Comparison of cofilin‐1 protein stability between young cells and old cells using densitometric quantification of blots. *: *p* < 0.05 for comparing the protein levels at the same time points. (c, e) Immunoprecipitation–immunoblot (IP–IB) assay for detection of the level of cofilin‐1 ubiquitination in WI‐38 cells, and in H_2_O_2_ treated A549 cells and H1299 cells. The WASP ubiquitination level was used as a negative control. The inputs included IB for cofilin‐1, WASP, GAPDH, and ubiquitin. For the ubiquitination assay, MG132 was used to pretreat WI‐38 cells (5 µM) and A549/H1299 cells (10 µM) for 8 hours, followed by IP‐IB. (f) Exogenous expression of 6xHis‐tag fused wild‐type cofilin‐1 and mutated Y68F cofilin‐1 in H1299 cells. The protein expression was detected by anti‐His‐tag antibody. (g) Staining of SA‐β‐gal after the over‐expression of wild‐type cofilin‐1 and mutant Y68F cofilin‐1. (h) Percentages of positive SA‐β‐gal stained cells (*N* = 100). *: *p* < 0.05

To investigate how cofilin‐1 was up‐regulated in senescent cells, we first examined the cofilin‐1 mRNA levels in replicative senescence and oxidative stress‐induced senescence. The RT‐qPCR showed that the cofilin‐1 mRNA levels were not significantly changed in the cell senescence induced by replication or oxidative stress compared with control cells (Figure S9). The stability of the cofilin‐1 protein was then examined in cells treated with protein translational inhibitor cycloheximide (CHX). Treatments ranged up to 32 hours, and samples were collected at different time points for the immunoblotting of cofilin‐1. This showed that the cofilin‐1 degradation rates of senescent WI‐38 cells and H_2_O_2_‐treated lung cancer cells were significantly slower than that of young WI‐38 cells and untreated cell controls, respectively (Figure 3a). These data were also quantified by densitometry (Figure 3b). To investigate whether the ubiquitination of cofilin‐1 was responsible for protein stability in cell senescence, an immunoprecipitation/immunoblot (IP‐IB) analysis was performed using an anti‐cofilin‐1 antibody (for IP), followed by an anti‐ubiquitin antibody (for IB). MG132, a 26S proteasome inhibitor used for the prevention of ubiquitylated protein degradation, was applied for the detection of the ubiquitylated form of the target proteins (Emmerich & Cohen, 2015). We randomly selected Wiskott–Aldrich syndrome protein (WASP), another actin‐associated protein involved in the regulation of actin dynamics, for comparison, because its expression was similar in young cells and senescent cells (Figure 1f). The results showed that the ubiquitination of cofilin‐1, but not that of WASP, was reduced in senescent cells compared with young cells (Figure 3c). Reduced cofilin‐1 ubiquitination was also detected in A549 cells and H1299 cells exposed to H_2_O_2_, but WASP ubiquitination was not significantly affected (Figure 3d and e). Furthermore, the over‐expression of WASP in WI‐38 neither induced SA‐β‐gal nor cofilin‐1 and several senescent biomarkers (Figure S10). It has been reported that the phosphorylation of tyrosine 68 (Y68) on the cofilin‐1 protein is required for protein degradation through the ubiquitin–proteasomal degradation pathway (Yoo et al., 2010). We therefore examined whether cofilin‐1 missing the phosphorylatable tyrosine could promote cell senescence. H1299 cells were transfected with His‐tagged wild‐type cofilin‐1 (cofilin‐WT) or phosphotyrosine mutant cofilin‐1 (cofilin‐Y68F), and the expression of these exogenous cofilin‐1 forms was detected using an anti‐His antibody (Figure 3f). The transfection efficiency of H1299 cells was about 80%, as determined by the transfection of pEGFP‐N1 plasmid (Figure S11). Both wild‐type cofilin‐1 and Y68F mutant cofilin‐1 increased the levels of SA‐β‐gal in H1299 cells compared with the vector transfected cells (Figure 3g). Y68F mutant cofilin‐1 also exhibited a stronger ability than wild‐type cofilin‐1 to induce SA‐β‐gal activity (Figure 3h). These results suggest that the accumulation of cofilin‐1 during cellular senescence is associated with an increase in protein stability.

### 2.4 Manipulation of cofilin‐1 expression could influence senescence‐related phenotypes

**Figure 4:**
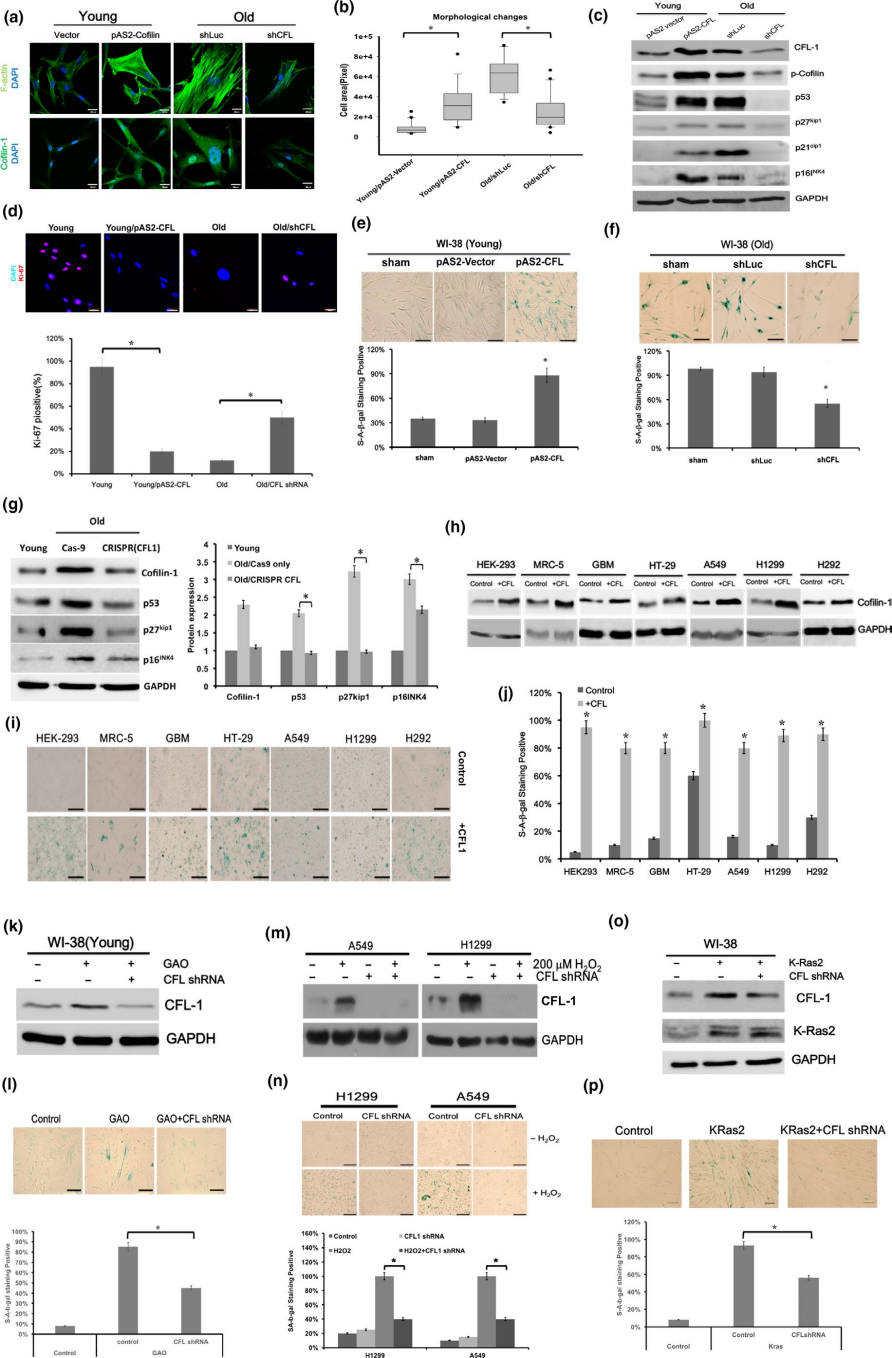
Manipulation of cofilin‐1 expression can regulate cell senescence. (a) Fluorescent staining of actin cytoskeleton in young WI‐38 cells and old WI‐38 cells, with or without manipulation of cofilin‐1. (b) Measurement of cell areas using the cell morphology analyzer (*N* = 25 for each group). (c) Over‐expression of cofilin‐1 (pAS2‐CFL1) and silencing of cofilin‐1 by CFL1 shRNA could up‐regulate and down‐regulate cell cycle inhibitors in WI‐38 cells, respectively. The empty pAS2 vector is the expressive control for pAS2‐CFL plasmid, and the shLuc construct is the off‐target control for CFL shRNA. (d) Detection of nuclear Ki‐67 in young WI‐38 cells and old WI‐38 cells, with or without manipulation of cofilin‐1. (e, f) Staining of SA‐β‐gal after the over‐expression of cofilin‐1 and silencing of cofilin‐1 in young cells and old cells, respectively. (g) CRISPR/Cas9 system‐mediated knockdown of the cofilin‐1 gene in old cells led to a reduced expression of p53, p27^Kip1^, and p16^INK4^, as determined by Western blot analysis and densitometry. (h) Transduction of pAS2‐CFL1 into various cell types, and the cofilin‐1 expression was detected by Western blot analysis. (i) Staining of SA‐β‐gal before and after the over‐expression of cofilin‐1 in different cell types. (j) Quantification of SA‐β‐gal positive stained cells before and after the over‐expression of cofilin‐1 in different cell types. (*N* = 100). (k) Silencing of cofilin‐1 in GAO‐treated WI‐38 cells. (l) The GAO induced SA‐β‐gal level was reduced by the silencing of cofilin‐1. (m) Silencing of cofilin‐1 in H_2_O_2_ treated A549 cells and H1299 cells. (n) H_2_O_2_ induced SA‐β‐gal level was reduced by the silencing of cofilin‐1. (o) Silencing of cofilin‐1 in K‐Ras2 transfected WI‐38 cells. (p) The K‐Ras2 induced SA‐β‐gal level was reduced by the silencing of cofilin‐1. SA‐β‐gal level represents the percentage of SA‐β‐gal expressing cells out of the total cells. Scale bar: 100 μm for bright field images and 30 μm for fluorescent images. *: *p* < 0.05. **: *p* < 0.01

To better understand whether cofilin‐1 expression is essential for the regulation of cell morphology and senescence, we over‐expressed and silenced cofilin‐1 in young and old cells, respectively, so as to examine the associated cell responses. The lentiviral‐based cofilin‐1 cDNA construct (pAS2‐CFL1) was transduced into young WI‐38 cells for the over‐expression of cofilin‐1. A cofilin‐1 shRNA construct (pLKO.1‐shCFL1) was used to infect old WI‐38 cells in order to silence the expression of cofilin‐1. The pAS2 empty vector was used as a negative control for pAS2‐CFL1 transduction, and the pLKO.1‐shLuc plasmid was used as an off‐target control for cofilin‐1 shRNA targeting experiments (Chang et al., 2012). First, the fluorescein‐conjugated phalloidin staining showed that stress fibers were increased by the over‐expression of cofilin‐1 in young cells, but were decreased by the knockdown of cofilin‐1 in old cells (Figure 4a). Using the pyrene‐conjugated actin polymerization assay, the cell lysates collected from the young WI‐38 cells transduced with cofilin‐1 cDNA could significantly decrease the actin depolymerization rate (Figure S12a). However, the cell lysate collected from the old WI‐38 cells transduced with cofilin‐1 shRNA weakly increased the actin depolymerization rate (Figure S12b). We also showed that the over‐expression of cofilin‐1 could increase the cell areas of the young cells, and the knockdown of cofilin‐1 could decrease that of the old cells (Figure 4b). Subsequently, we assessed the expression of the cell cycle inhibitors involved in senescence‐associated growth arrest. We found that the over‐expression of cofilin‐1 in young cells could induce p53, p21^Cip1^, p27^Kip1^, p16^INK4^, and p‐cofilin‐1, but the silencing of cofilin‐1 in senescent cells suppressed these molecules (Figure 4c). The over‐expression of cofilin‐1 in young cells also suppressed Ki‐67, a marker of proliferation, to a level similar to that of old cells, and the silence of cofilin‐1 in old cells could partially rescue the expression of Ki‐67 (Figure 4d). We then found that the levels of SA‐β‐gal stained cells were increased by the over‐expression of cofilin‐1 in young cells and were decreased by the knockdown of cofilin‐1 in senescent cells (Figure 4e and f). Additionally, the over‐expression of cofilin‐1 suppressed the growth rates of young WI‐38 cells (see below, Figure 6o). However, the knockdown of cofilin‐1 could recover cell growth in old cells, but suppress cell growth in young cells (Figure S13a and b). We also found that losses of lamin B1 and actin‐interacting protein 1 (Aip1) in senescent cells were partially rescued, and the expression of the senescence‐associated marker γH2AX was reduced after the knockdown of cofilin‐1 (Figure S13c). We also transduced the CRISPR/Cas‐9 gene editing system to knockdown cofilin‐1 gene in old cells. This system worked as expected, in that the cofilin‐1 expression in old cells was reduced and accompanied by the down‐regulation of p53, p27^Kip1^, and p16^INK4^ compared with the Cas9 only control (Figure 4g). Moreover, the growth ratio of the CRISPR/Cas‐9 infected old cells was greater than that of the control cells for up to four days in the culture (Figure S13d). To investigate whether the over‐expression of cofilin‐1 could promote senescence in various cell types, a pAS2‐CFL construct was transduced into various cell lines. These included HEK293, MRC‐5, GBM (S1R1; (Lin et al., 2013), HT‐29, A549, H1299, and H292 cells (Figure 4h). Interestingly, the expression of cofilin‐1 cDNA in these cell lines led to increases in SA‐β‐gal activity (Figure 4i). These results were further quantified to demonstrate that cofilin‐1 could increase the percentage of positive SA‐β‐gal stained cells in different cell types compared with vector transfected controls (Figure 4j).

The effects of cofilin‐1 on oxidative stress‐induced senescence were further examined. WI‐38 cells were transduced with cofilin‐1 shRNA to silence cofilin‐1 expression and then treated with GAO to induce endogenous H_2_O_2_ (Figure 4k). It appeared that the increased SA‐β‐gal level induced by GAO could be suppressed by silencing cofilin‐1 (Figure 4l). We also transduced cofilin‐1 shRNA into A549 cells and H1299 cells treated with H_2_O_2_ (Figure 4m), and the induction of the SA‐β‐gal level by H_2_O_2_ was suppressed by the knockdown of cofilin‐1 (Figure 4n). Furthermore, cofiin‐1 was silenced in the cells transduced with the K‐Ras2 oncogene (Figure 4o), and the SA‐β‐gal level induced by K‐Ras2 was repressed (Figure 4p). Taken together, these data suggest that cofilin‐1 is not only a potent senescent marker, but is also involved in regulating cell senescence.

### 2.5 Effects of p27^Kip1^ on mediating cell senescence caused by cofilin‐1

**Figure 5:**
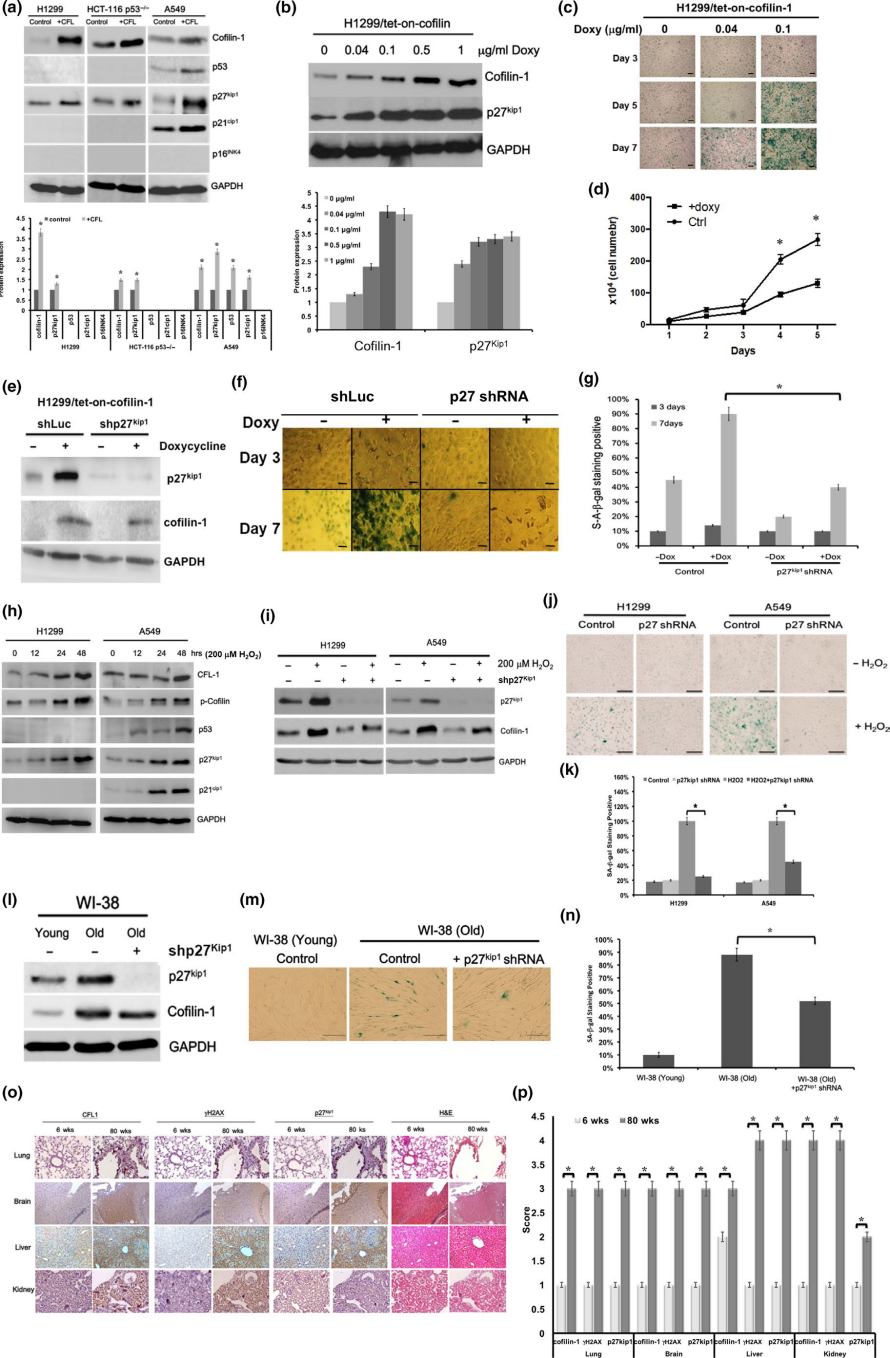
P27^Kip1^ is essential for the over‐expression of cofilin‐1 promoted cell senescence. (a) Up‐regulation of p27^Kip1^ was detected in cells transduced with cofilin‐1, with or without p53 and p16^INK4^ expression. (b) Expression of p27^Kip1^ was accompanied by the induction of cofilin‐1 in H1299/tet‐on‐cofilin‐1 cells treated with dose‐dependent doxycycline. (c) Time‐dependent expression of SA‐β‐gal in H1299/tet‐on‐cofilin‐1 cells correlated to the dose‐dependent expression of cofilin‐1. (d) Comparison of growth rates between control and doxycycline treated H1299/tet‐on‐cofilin‐1 cells. Doxycycline: 0.1 µg/ml. (e) Silencing of p27^Kip1^ in cofilin‐1 over‐expressing H1299/tet‐on‐cofilin‐1 cells using shRNA. Doxycycline: 0.1 µg/ml. (f) Silencing of p27^Kip1^ reduced the level of SA‐β‐gal staining in cofilin‐1 over‐expressing cells. Scale bar: 100 µm. (g) Quantification of SA‐β‐gal stained cells after the knockdown of p27^Kip1^ before and after the over‐expression of cofilin‐1. (h) H_2_O_2_ treatment induced cofilin‐1 and p27^Kip1^ in H1299 (p53‐/‐) cells and A549 (p53+/+) cells. (i) Silencing of p27^Kip1^ in H_2_O_2_ (200 μM) treated H1299 cells and A549 cells. (j) Staining of SA‐β‐gal in H1299 and A549, with or without silencing of p27^Kip1^, followed by H_2_O_2_. Scale bar: 100 μm. (k) Quantification of SA‐β‐gal staining in p27^Kip1^ silencing cells treated with H_2_O_2_. (l) Silencing of p27^Kip1^ in senescent WI‐38 cells using shRNA. (m) SA‐β‐gal staining for senescent WI‐38 cells, before and after the silencing of p27^Kip1^. (n) Quantification of SA‐β‐gal staining in p27^Kip1^ silencing WI‐38 cells. (o) IHC detection of cofilin‐1, p27^Kip1^, and γH2AX expressions in different tissues obtained from 6‐week‐old and 80‐week‐old mice (*N* = 3 for each group). H&E staining was used for the histological diagnosis. Scale bar: 50 μm. (p) Quantification of IHC score of (o). *: *p* < 0.05

Although the over‐expression of cofilin‐1 directly induces cell senescence, the underlying mechanisms remain unclear. Because the up‐regulation of cofilin‐1 was accompanied by increased p53, p21^Cip1^, p27^Kip1^, and p16^INK4^ in replicative senescence, we examined which of these could be directly regulated by cofilin‐1. We over‐expressed cofilin‐1 in three cell lines with different p53 and p16^INK4^ statuses—H1299 (p53^−/−^; p16^−/−^), HCT116 (p53^−/−^; p16^−/−^), and A549 cells (p53^+/+^; p16^−/−^)—and showed that p27^Kip1^ could be up‐regulated in these cell lines, regardless of p53 and p16^INK4^ status (Figure 5a). Previously, we established stable H1299/*tet*‐*on*‐cofilin‐1 cells that could be induced to express cofilin‐1 via doxycycline treatment (Tsai et al., 2009). In these cells, the doxycycline‐induced expression of cofilin‐1 led to the up‐regulation of p27^Kip1^ in a dose‐dependent manner (Figure 5b). The expression of p21^Cip1^ was not induced by the over‐expression of cofilin‐1 in H1299/*tet*‐on‐cofilin‐1 cells (Figure S14). The SA‐β‐gal levels were increased upon the induction of cofilin‐1, for up to seven days of incubation (Figure 5c). Concomitantly, the growth rate was also reduced upon the over‐expression of cofilin‐1 in these cells (Figure 5d). To determine whether p27^Kip1^ expression is important for this effect, p27^Kip1^ was silenced in cells over‐expressing cofilin‐1 (Figure 5e). The SA‐β‐gal staining, promoted by the over‐expressed cofilin‐1 in H1299/*tet*‐*on*‐cofilin‐1 cells, could be suppressed by silencing p27^Kip1^ (Figure 5f and g). Moreover, we also silenced p27^Kip1^ in cofilin‐1 over‐expressed young WI‐38 cells and showed that the induced SA‐β‐gal was reduced rather than the knockdown of p21^Cip1^ and p16^INK4^ in this condition (Figure S15). The cofilin‐1‐p27^Kip1^ axis for cell senescence was further investigated in oxidative stress‐induced senescence. Both A549 cells and H1299 cells were treated with H_2_O_2_ for up to 48 hours. The results showed that p27^Kip1^ was induced in both cell lines in a time‐dependent manner; however, p21^Cip1^ was only induced in A549 cells with a normal p53 activity (Figure 5h). The blots were quantified by densitometry (Figure S16). We silenced p27^Kip1^ in both the H1299 cells and A549 cells, followed by H_2_O_2_ treatment to examine whether the SA‐β‐gal staining would be reduced. The knockdown of p27^Kip1^ was efficient in both cell types, with or without H_2_O_2_ treatment (Figure 5i). Furthermore, the knockdown of p27^Kip1^ reduced the H_2_O_2_‐induced SA‐β‐gal activity (Figure 5j and k). To confirm whether p27^Kip1^ is also involved in senescent cells, we silenced p27^Kip1^ in old WI‐38 cells (Figure 5l). The knockdown of p27^Kip1^ could reduce the level of SA‐β‐gal staining in old cells (Figure 5m). Although this effect is significant, the SA‐β‐gal activity of old cells after the knockdown of p27^Kip1^ remained higher than that of the young cells (Figure 5n). Finally, we examined whether cofilin‐1 and p27^Kip1^ were co‐expressed in different tissues of aged mice using IHC. Consecutive cryosections of lung, brain, liver, and kidney tissues showed that cofilin‐1 was co‐expressed with p27^Kip1^ and another marker of senescence, γH2AX (Figure 5o). The scoring of the IHC results showed that these molecules were up‐regulated in aged tissues compared with young tissues (Figure 5p).

### 2.6 Effects of TEAD1 on the regulation of p27^Kip1^ gene expression during cell senescence

**Figure 6:**
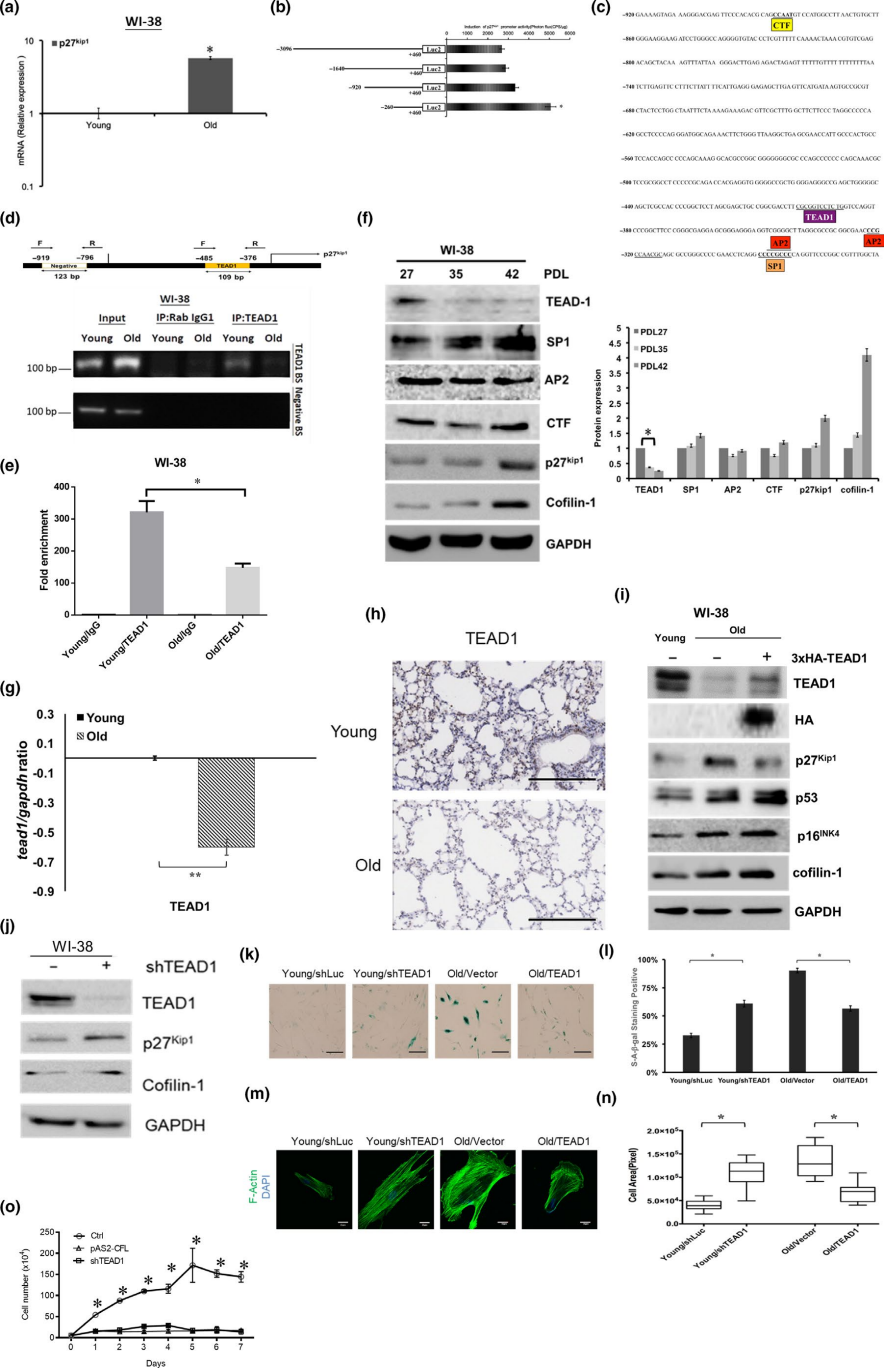
Transcriptional enhancer factors domain one (TEAD1) represses p27^Kip1^ gene expression to regulate cell senescence. (a) RT‐qPCR showed the increase of p27^Kip1^ mRNA in senescent WI‐38 cells. (b) Luciferase reporter gene assay for the sequence deletion of the p27^Kip1^ gene promoter constructs performed in HEK293 cells. (c) Use of the Transcriptional Regulatory Element Database (TRED) database for the prediction of the putative binding sites of the transcription factors. (d) ChIP assay for determining the binding of TEAD1 on the putative binding sequence (TEAD1 BS) of p27^Kip1^ gene promoter of young cells and old cells. A negative binding site (negative BS) selected between −796 bp to −919 bp, without a putative TEAD1 binding sequence. (e) A Chromatin immunoprecipitation (ChIP)‐qPCR assay for comparison of the fold enrichment of TEAD1 bound to the p27^Kip1^ gene promoter of young cells and old cells. (f) The expressions of the TEAD1, SP1, AP2, and CTF transcription factors were examined with the expression of cofilin‐1 and p27^Kip1^ in the WI‐38 cells with increased PDLs. (g) TEAD1 mRNA level was decreased in senescent cells compared with young cells. (h) IHC staining of TEAD1 protein in lung tissue sections obtained from young mice and old mice. (i) Effects of the over‐expression of TEAD1 in senescent WI‐38 cells on the expression of p27^Kip1^, p53, p16^INK4^, and cofilin‐1. (j) Effects of TEAD1 silencing on young WI‐38 cells. The expressions of p27^Kip1^ and cofilin‐1 are examined. (k) Effects of TEAD1 silencing on the cell growth of young cells. (l) The level of SA‐β‐gal increased after the knockdown of TEAD1 in young cells, and decreased after the over‐expression of TEAD1 in old cells. (m) Visualization of actin cytoskeleton in TEAD1 silenced young cells and TEAD1 over‐expressed old cells using fluorescein‐conjugated phalloidin staining. (n) Measurement of cell areas. *N* = 15 for each group. Scale bar: 100 μm for bright field images and 30 μm for fluorescent images. (o) Effects of the knockdown of cofilin‐1 and over‐expression of cofilin‐1 on the suppression of cell proliferations. *: *p* < 0.05

Compared with young cells, we found that the p27^Kip1^ mRNA levels were increased in senescent cells (Figure 6a). To explore how the p27^Kip1^ gene was transcribed, we examined its promoter activity. Four reporter constructs with a series of deleted promoter sequences were separately subcloned to a pGL4.1‐Luc2 vector. The vector was then transfected into HEK293 cells to evaluate the promoter activity by luciferase assay. Interestingly, the shortest promoter of the construct (–260 bp) exhibited the highest luciferase activity, relative the full‐length promoter construct (Figure 6b). A survey of the sequence between −920 bp and −260 bp using the Transcriptional Regulatory Element Database (TRED) found four potent transcription factor binding elements, including CCAAT box‐binding transcription factor (CTF), TEAD1, activating protein 2 (AP2), and Sp1 transcription factor (Figure 6c). To explore if TEAD1 could differentially interact with the putative binding site on the p27^Kip1^ gene promoter in young cells compared with old cells, the ChIP assay was used. Primers were designed to amplify a 109 bp product from a TEAD1 binding site on the p27^Kip1^ gene promoter (see Materials and Methods). The results showed that a PCR product with the expected size was amplified in young cells, but not in old cells after ChIP, using the anti‐TEAD1 antibody, and no PCR product could be visualized by amplifying a distal negative binding site lacking a TEAD1 binding sequence (Figure 6d). A ChIP‐qPCR experiment was also conducted to confirm that the fold enrichment of the interaction between TEAD1 and the p27^Kip1^ gene promoter was higher in young cells than in old cells (Figure 6e). Next, we showed that the TEAD1 levels, but not the other transcription factors mentioned above, were significantly reduced in higher PDL WI‐38 cells (Figure 6f). TEAD1 was also down‐regulated in senescent cells (senescence was induced by K‐Ras2 oncogene and H_2_O_2_) with up‐regulated cofilin‐1 (Figure S17). A reduction of TEAD1 mRNA was also detected in old cells compared with young cells (Figure 6g). A down‐regulation of TEAD1 was found in lung tissue sections of old mice using the IHC staining (Figure 6h). We next investigated whether the manipulation of TEAD1 could influence the expression of p27^Kip1^ and senescent phenotypes. The restoration of TEAD1 by the transduction of the 3xHA‐TEAD1 construct into senescent cells could suppress p27^Kip1^, but not p53, p16^INK4^, or cofilin‐1 (Figure 6i). Additionally, the knockdown of TEAD1 could increase the p27^Kip1^ expression in young WI‐38 cells (Figure 6j). SA‐β‐gal staining showed that the knockdown of TEAD1 could increase the SA‐β‐gal activity in young cells, and the over‐expression of TEAD1 could reduce that in old cells after quantification (Figure 6k and l). The knockdown of TEAD1 in young cells increased the stress fibers and cell areas, and the over‐expression of TEAD1 reversed these phenomena (Figure 6m and n). The knockdown of TEAD1 exhibited similar effects with an over‐expression of cofilin‐1 on the suppression of the growth rate of young cells (Figure 6o). The over‐expression of TEAD‐1 in old cells showed an increase in the growth ratio compared with the untransduced controls (Figure S18). Taken together, these data suggest that during cell senescence, TEAD1 can regulate p27^Kip1^ at the transcriptional level.

### 2.7 Cofilin‐1 mediates the expression of TEAD1 to regulate p27^Kip1^ and cell senescence

**Figure 7:**
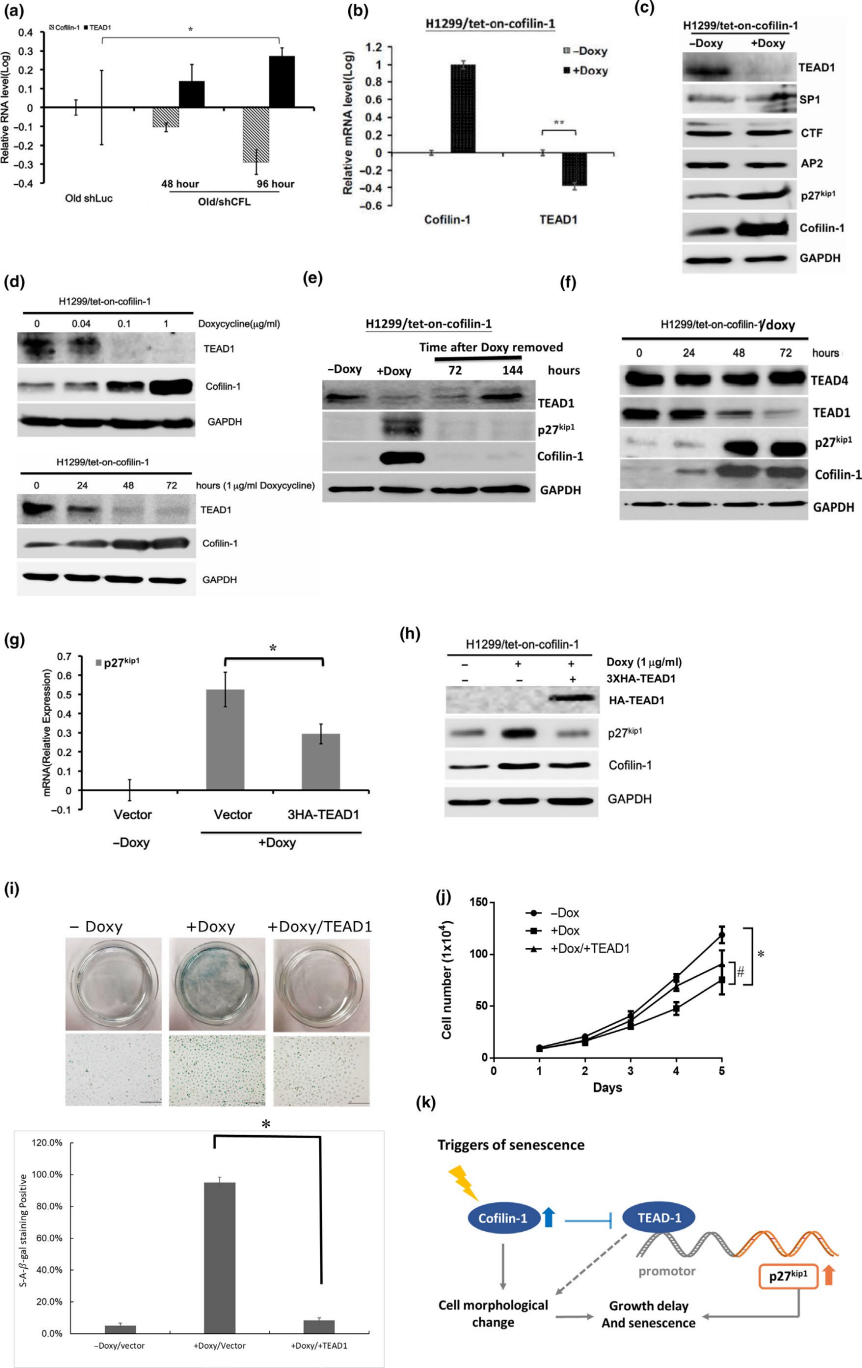
Cofilin‐1 negatively regulates TEAD1 to mediate the expression of p27^Kip1^ and cell senescence. (a) The knockdown of cofilin‐1 in old cells recovered the expression of TEAD1 mRNA using RT‐qPCR. (b) Over‐expression of cofilin‐1 in H1299/tet‐on‐cofilin‐1 cells suppressed the expression of TEAD1 mRNA. (c) Over‐expression of cofilin‐1 in H1299/*tet*‐*on*‐cofilin‐1 cells suppressed the expression of the TEAD1 transcription factor. (d) Dose‐dependent and time‐dependent suppression of TEAD1 in H1299/*tet*‐*on*‐cofilin‐1 cells treated with doxycycline for cofilin‐1 induction. The cells were treated with doxycycline for 48 hours in the dose‐dependent experiment. (e) Removal of doxycycline in the doxycycline treated H1299/*tet*‐*on*‐cofilin‐1 cells led to a reduction of cofilin‐1, followed by a recovery of TEAD1 expression. Cells were treated with 0.1 µg/ml of doxycycline for 36 h and then replaced by a normal medium. (f) Over‐expression of cofilin‐1 suppresses the expression of TEAD1, but not that of TEAD4. (g) p27^Kip1^ mRNA and (h) p27^Kip1^ protein induced by over‐expressed cofiin‐1 was suppressed by the transduction of the 3xHA‐TEAD1 construct. (i) SA‐β‐gal staining showed that the over‐expression of cofilin‐1 induced cell senescence was suppressed by the restoration of TEAD1. Scale bar: 200 µm. (j) Comparison of cell growth rates using the hemocytometry. (k) Illustration of the potent cofilin‐1/TEAD1/p27^Kip1^ regulatory pathway for cell senescence. *, #: *p* < 0.05; **: *p* < 0.01

To investigate whether the down‐regulation of TEAD1 mRNA is associated with up‐regulated cofilin‐1 in senescent cells, we silenced cofilin‐1 and found that the TEAD1 mRNA levels were restored in senescent cells (Figure 7a). Additionally, the over‐expression of cofilin‐1 could suppress the expression of TEAD1 transcripts and protein in H1299/*tet*‐*on*‐cofilin‐1 cells (Figure 7b and c). On the other hand, the Sp1, CTF, and AP2 transcription factors that might bind to the p27^Kip1^ gene promoter were not affected by the over‐expression of cofilin‐1 (Figure 7c). Dose‐dependent and time‐course suppression of TEAD1 by over‐expressed cofilin‐1 were also detected in this cell model (Figure 7d). The removal of doxycycline in these cells led to the recovery of the cofilin‐1 level, followed by the restoration of the TEAD1 and p27^Kip1^ levels (Figure 7e). Furthermore, the over‐expression of cofilin‐1 only suppressed TEAD1, not TEAD4 (Figure 7f). To determine whether TEAD1 is a mediator of cofilin‐1 induced p27^Kip1^ and senescence, we transduced a 3xHA‐TEAD1 construct into cofilin‐1 over‐expressing cells. The results showed that the induction of p27^Kip1^ mRNA by the over‐expression of cofilin‐1 was suppressed by transduced TEAD1 cDNA (Figure 7g). These effects were also detected at the protein level (Figure 7h). The induction of the SA‐β‐gal level by the over‐expression of cofilin‐1 was also suppressed by the restoration of TEAD1 in these cells (Figure 7i). The restoration of TEAD1 in cofilin‐1 over‐expressing H1299/*tet*‐*on*‐cofilin‐1 cells could also recover delayed cell growth (Figure 7j). Cell senescence was not affected by the transduction of TEAD1 alone (data not shown). Taken together, a putative cofilin‐1/TEAD1/p27^Kip1^ regulatory axis involved in the morphological change and growth delay of cell senescence is illustrated (Figure 7k).

## 3 DISCUSSION

In cell senescence, morphological change correlates with increased rigid cytoskeletal structures formed by actin filaments, microtubules, and intermediate filaments. For microtubules, the hyperphosphorylation of microtubule‐associated protein tau has been found in senescence‐accelerated mice (Canudas et al., 2005). The cross‐bridging protein p50 is also reported to form large bundles of intermediate filaments in senescent fibroblasts (Wang, 1985). On the other hand, the involvement of actin‐associated proteins is less reported in cell senescence (Hernandez‐Segura et al., 2018). Our current data show that the actin depolymerization rate was significantly reduced with an increase of phosphorylated cofilin‐1, as demonstrated by Western blot and 2D gel blot assays. The over‐expression of wild‐type cofilin‐1 and mutant S3D cofilin‐1 induced similar changes of actin re‐organization, cell size, and SA‐β‐gal activity, suggesting that cofilin‐1 phosphorylation is involved in cell senescence. On the contrary, the over‐expression non‐phosphorylatable S3A mutant cofilin‐1 did not influence the actin cytoskeleton and cell morphology. Although S3A mutant cofilin‐1 increased the SA‐β‐gal activity in transduced cells, the level was lower than wild‐type cofilin‐1 and mutant S3D cofilin‐1 transduced cells. The fact that the over‐expression of S3A mutant cofilin‐1 could induce a senescent phenotype is not a surprise, because it has been reported that the nuclear accumulation of globular actin and dephosphorylated cofilin occur in cell senescence (Kwak et al., 2004). Therefore, current data suggest that both phosphorylated cofilin‐1 and dephosphorylated cofilin‐1 could contribute to cell senescence and growth delays, but through different pathways. The significance of cofilin‐1 phosphorylation in cell senescence may be also investigated by the manipulation of cofilin‐specific kinases or phosphatases.

The up‐regulation of cofilin‐1 is not only detected in replicative senescence, but also in oxidative stress‐induced senescence and oncogene‐induced senescence. Oxidative stress and oncogene activation are known to promote carcinogenesis, but they also induce senescence to create negative feedback loops in tumor development via the p53/p21^Cip1^ and p16^INK4^/RB tumor suppressive pathways (Mijit et al., 2020; Prieur et al., 2011). An assessment of the percentage of SA‐β‐gal positive cells over the total cells showed that the knockdown of cofilin‐1 could reduce oxidative stress‐induced and oncogene‐induced senescence. This suggests that the up‐regulation of cofilin‐1 may also be involved in the anti‐proliferative effects caused by these growth stresses.

In addition to cultured cells, we also demonstrated that cofilin‐1, but not ADF, was up‐regulated in the lung tissue of aged mice. It has been reported that cofilin‐1 and ADF are differentially expressed in various tissues of adult mice (Gurniak et al., 2005). Through the IHC staining, we did not detect a significant difference of ADF expression in the lung tissue of young mice or that of old mice (Figure S19a). The over‐expression of ADF in WI‐38 cells also did not influence the expression of cofilin‐1, p53, p27^Kip1^, p16^INK4^, and cell morphology (Figure S20b and c). It seems possible that cofilin‐1, but not ADF, would regulate cell senescence. The expression of cofilin‐1 in aged tissues may be associated with pathophysiological events. For instance, the cofilin‐1 level was increased in the urine collected from patients with age‐related ischemic shock and acute kidney injury (Chao et al., 2012). Additionally, increased brain cofilin‐1 was observed in a Tg19959 mouse model of Alzheimer's disease at only 16 weeks of age (Yao et al., 2010). Therefore, cofilin‐1 may be considered a potent biomarker of senescence‐related diseases.

Cell rejuvenation remains a challenging topic, because replicative senescence is caused by telomere erosion and irreversible growth arrest (Rodier & Campisi, 2011). We have demonstrated the shortening of telomeres in high PDLs of WI‐38 cells. As a novel senescence‐related molecule, the knockdown of cofilin‐1 reduced the stress fibers and cell size of senescent cells, accompanied by decrease in the surrogate SA‐β‐gal marker. These observations were consistent with the increased growth rate of old cells after the knockdown of cofilin‐1. Another interesting finding is the reduction of Aip1 in senescent WI‐38 cells. Aip1 can promote the severing of actin filaments by cofilin‐1 and is a cofactor of cofilin‐1 to enhance actin dynamics (Chen et al., 2015; Chu et al., 2012). Because the actin depolymerization rate was significantly slowed in senescent cells, increased cofilin‐1 (at phosphorylated form) and decreased Aip1 could be sufficient to explain this phenomenon. Little is known if Aip1 is involved in the senescence of mammalian cells, although a robust knockdown of Aip1 can lead to cell senescence in plants (Augustine et al., 2011). Our study suggests that Aip1 is involved in cofilin‐1‐mediated actin re‐organization and morphological change in cell senescence.

The involvement of p27^Kip1^ in cofilin‐1‐mediated G0/G1 phase arrest has been reported (Tsai et al., 2009; Wang et al., 2016). Here, we further showed that cofilin‐1 induced p27^Kip1^ was essential for cell senescence caused by various stresses when p53 and p16^INK4^ were null. The cofilin‐1/p27^Kip1^ signaling pathway may be parallel to the p53/p21^Cip1^ and Rb/p16^INK4^ pathways in regulation of cell senescence, because the knockdown of p27^Kip1^ does not fully reverse the senescent related phenotypes in cells with a normal p53 and/or p16^INK4^. A significant role of p16^INK4^ in the p53‐independent promotion of senescence has been reported, and the bypass of telomere attrition directed senescence could be nearly detected by the combined inhibition of p16^INK4^ and p53 (Jacobs & de Lange, 2004). Thus, it implies that other cell cycle regulated mechanisms may be also independently involved in the development of cell senescence. In this study, although the manipulation of cofilin‐1 could influence the expression of p53, p27^Kip1^, p21^Cip1^, and p16^INK4^ in primary cells, the use of p53/p16^INK4^‐null cell lines demonstrated that p27^Kip1^ was mainly ablated by cofilin‐1, because p21^Cip1^ was not affected by the over‐expression of cofilin‐1. P53 and p16^INK4^ are known as tumor suppressor genes, and their inactivation can render risks of tumorigenesis (Romagosa et al., 2011; Schmitt et al., 2002). Unlike p53 and p16^INK4^, the mutation or deletion of p27^Kip1^ is rarely found in human cancers, although the expression would be dysregulated through level reduction (Slingerland & Pagano, 2000). Therefore, the elevation of p27^Kip1^ may be interesting to design a strategy for tumor control. An example is that the screening of Skp2 E3 ligase inhibitors is to increase the p27^Kip1^ stability and prevent cancer growth (Wu et al., 2012). Thus, activation of the cofilin‐1/p27^Kip1^ pathway may be related to tumor control.

The p27^Kip1^ gene promoter has been previously cloned, and a series of promoter deletion analyses have been studied (Minami et al., 1997). We then found a putative TEAD1 binding site on the proximal position of the promoter and showed that TEAD1 could bind to the p27^Kip1^ gene promoter in young cells, but not in senescent cells, using the ChIP‐qPCR assay. We also found that the knockdown of TEAD1 was sufficient to induce p27^Kip1^, but not cofilin‐1, and the over‐expression of TEAD1 could suppress p27^Kip1^ in cofilin‐1 over‐expressing cells. Therefore, TEAD1 may function as a transcriptional repressor to regulate the p27^Kip1^ gene expression. TEAD1 has been found to repress smooth muscle‐specific gene expression by binding to myocardin (Liu et al., 2014). TEAD1 also showed transrepressive activity on the promoters of the prolactin gene and human chorionic somatomammotropin (hCS) gene (Jiang & Eberhardt, 1996; Kessler et al., 2008). To the best of our knowledge, this is the first report showing that TEAD1 could bind to the promoter region of the p27^Kip1^ promoter. However, we also found that the restoration of TEAD1 in senescent diploid fibroblasts could only suppress the p27^Kip1^ level, but not p53 and p16^INK4^. Therefore, TEAD1 should specifically target p27^Kip1^, but may only account for one of the senescent mechanisms. Moreover, the manipulation of TEAD1 alone could affect both the cell morphology and SA‐β‐gal level. On the contrary, the manipulation of p27^Kip1^ did not change the cell morphology, but only influenced the SA‐β‐gal levels of young and old cells (Figure S20). Therefore, cofilin‐1, TEAD1, and p27^Kip1^ may affect different properties of cell senescence, although they work conjunction in an axis.

In summary, we found that cofilin‐1 was post‐translationally accumulated in cell senescence. Additionally, cofilin‐1 could induce p27^Kip1^ for cell growth arrest via the negative regulation of the TEAD1 transcription factor. Because up‐regulated cofilin‐1 mainly existed in serine‐3 phosphorylated form, this might explain the increased stress fibers and cell areas in senescent cells. However, several limitations and potent questions still need to be addressed. First, whether the kinases and phosphatases involved in the regulation of cofilin‐1 phosphorylation will also influence cell senescence? Second, the most interesting question is how cofilin‐1 regulates the expression of TEAD1 mRNA? As cofilin‐1 has been reported to elongate RNA polymerase II transcription (Obrdlik & Percipalle, 2011), it seems plausible that cofilin‐1 will regulate gene expression, including TEAD1. Finally, a recent report indicates that YAP/TEADs can up‐regulate S‐phase kinase‐associated protein 2 (Skp2) SCF ubiquitin ligase to degrade p27^Kip1^ for G0 exit (Jang et al., 2017). Although we found TEAD1 could transrepress p27^Kip1^ gene transcription in cell senescence, it is still an open question as to whether cofilin‐1 could regulate p27^Kip1^ through the YAP/TEADs‐Skp2 pathway. Besides, the cell culture system was another limitation that a few of non‐senescent cells in the cell population might be still growing rather than manipulated by cofilin‐1. Future studies should consider the effects of over‐expressed cofilin‐1 in the single cell. Taken together, the cofilin‐1/TEAD1/p27^Kip1^ regulatory axis may be a novel senescence‐associated signaling pathway for the regulation of the cell morphology and growth.

## CONFLICT OF INTEREST

The authors declare no conflict of interest.

## AUTHOR CONTRIBUTIONS

CHT and CYC contributed to investigation, data acquisition and curation, formal analysis, and visualization. BZL, YLW, and MHW contributed to experimental performance and acquisition of data. LTL contributed to experimental design and visualization. WCH contributed to resources. JDH and TJS contributed to editing of manuscript and conception. JSL contributed to software. RNK contributed to conception. PHT contributed to formal analysis. YJL contributed to conception, experimental design, supervision, visualization, writing and editing of original manuscript, funding acquisition, and project administration.

## Supporting information

Figure S1

Figure S2

Figure S3

Figure S4

Figure S5

Figure S6

Figure S7

Figure S8

Figure S9

Figure S10

Figure S11

Figure S12

Figure S13

Figure S14

Figure S15

Figure S16

Figure S17

Figure S18

Figure S19

Figure S20

Table S1

Table S2

Supporting information

## Data Availability

The data that support the findings of this study are available from the corresponding author upon reasonable request.
