# Supplementary figures and images for: Up‐regulation of cofilin‐1 in cell senescence associates with morphological change and p27kip1‐mediated growth delay
Source: Aging Cell. 2020 Dec 18;20(1):e13288. doi: 10.1111/acel.13288 (PMC7811848; doi:10.1111/acel.13288)

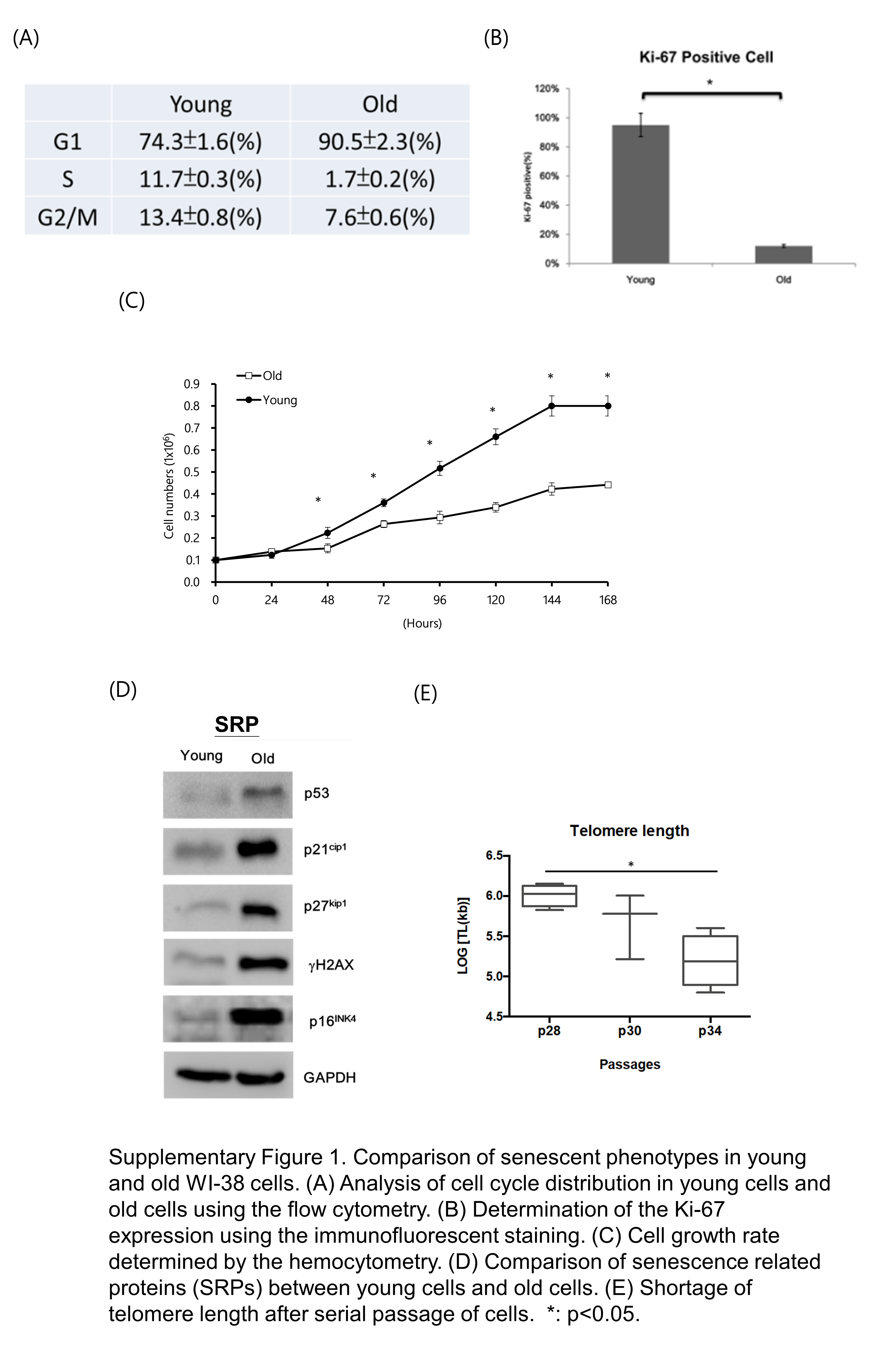

Supplement: Supplementary file 1 — Figure S1 [file ACEL-20-e13288-s001.TIF]

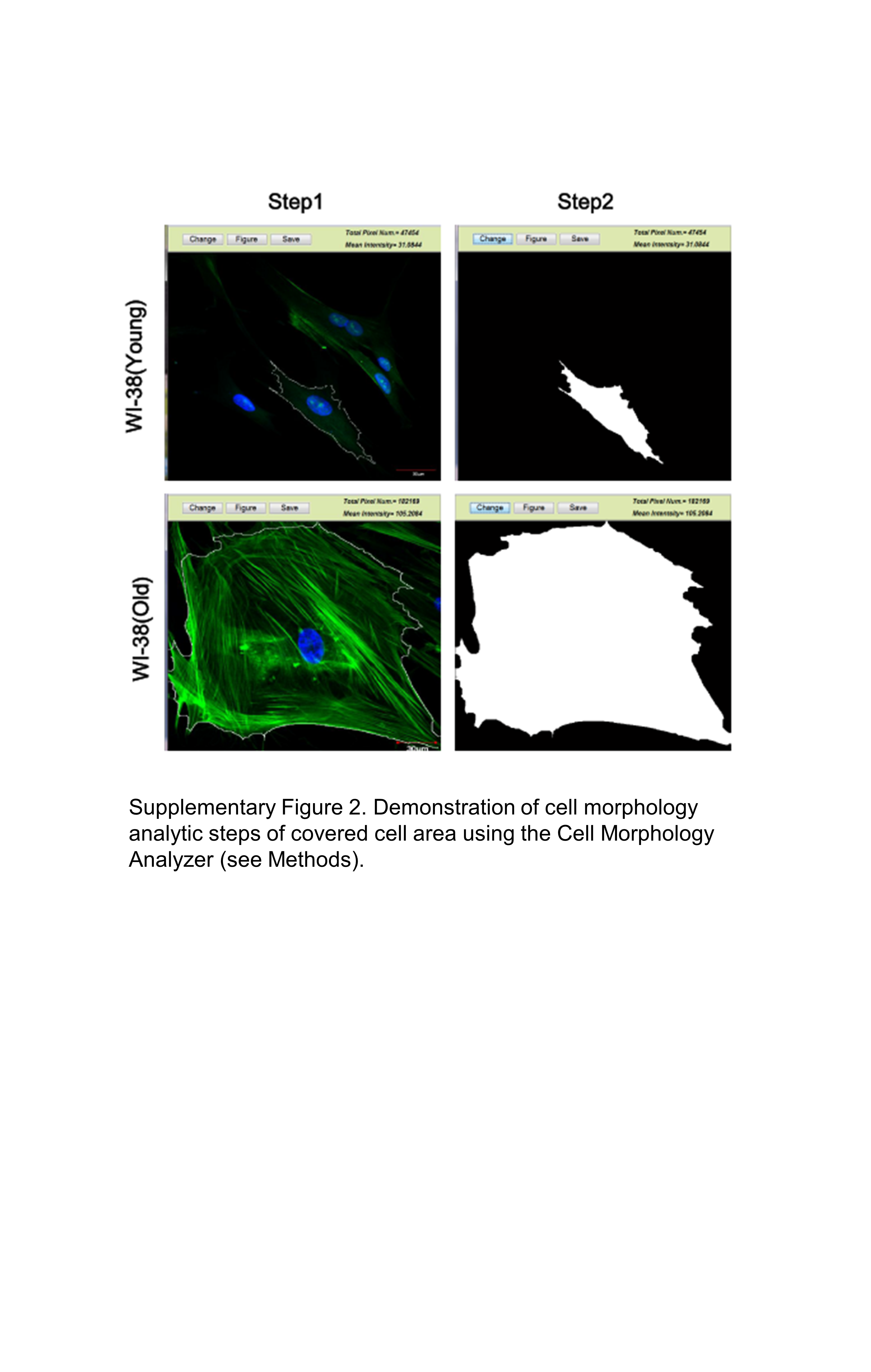

Supplement: Supplementary file 2 — Figure S2 [file ACEL-20-e13288-s002.TIF]

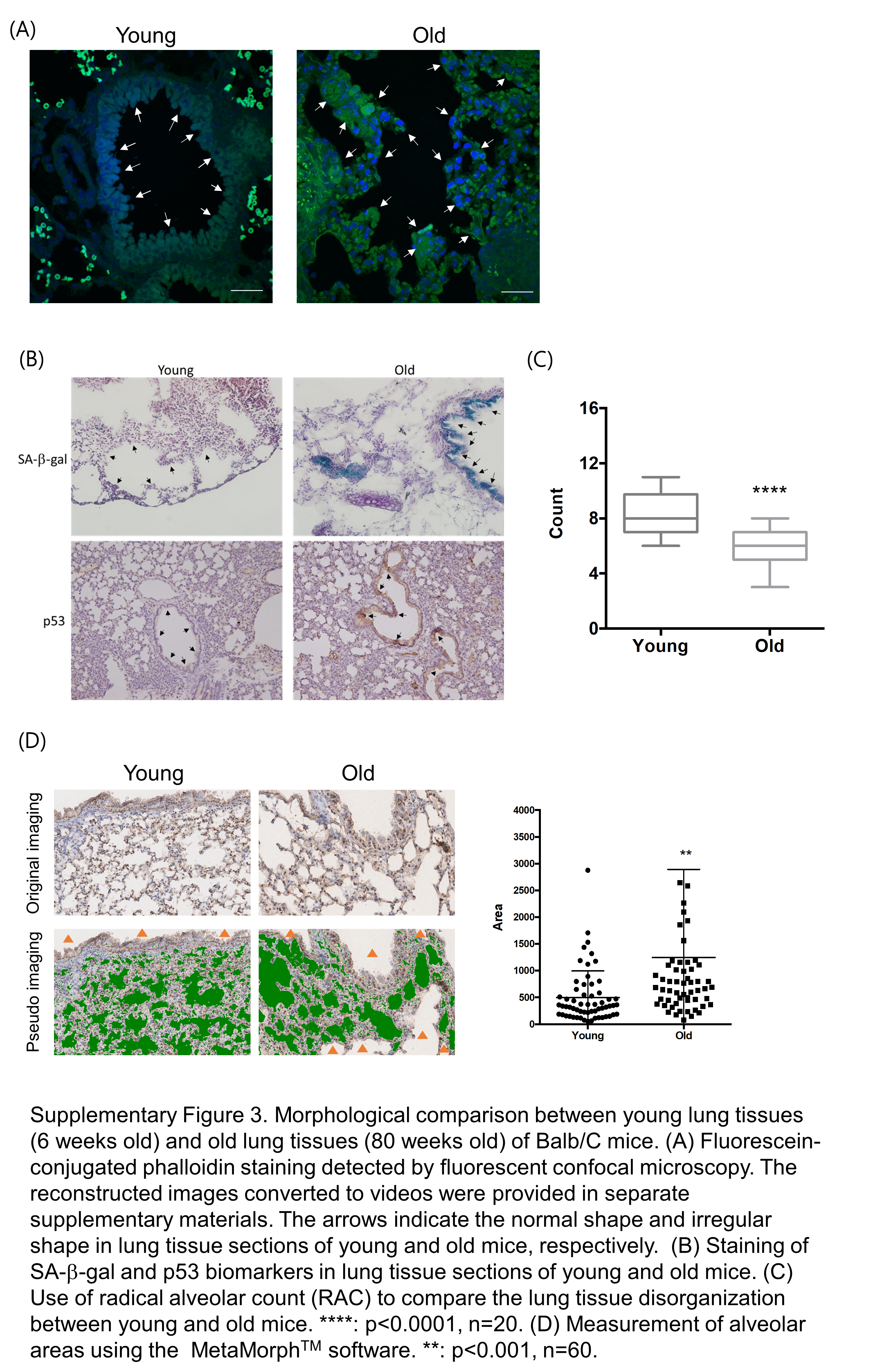

Supplement: Supplementary file 3 — Figure S3 [file ACEL-20-e13288-s003.TIF]

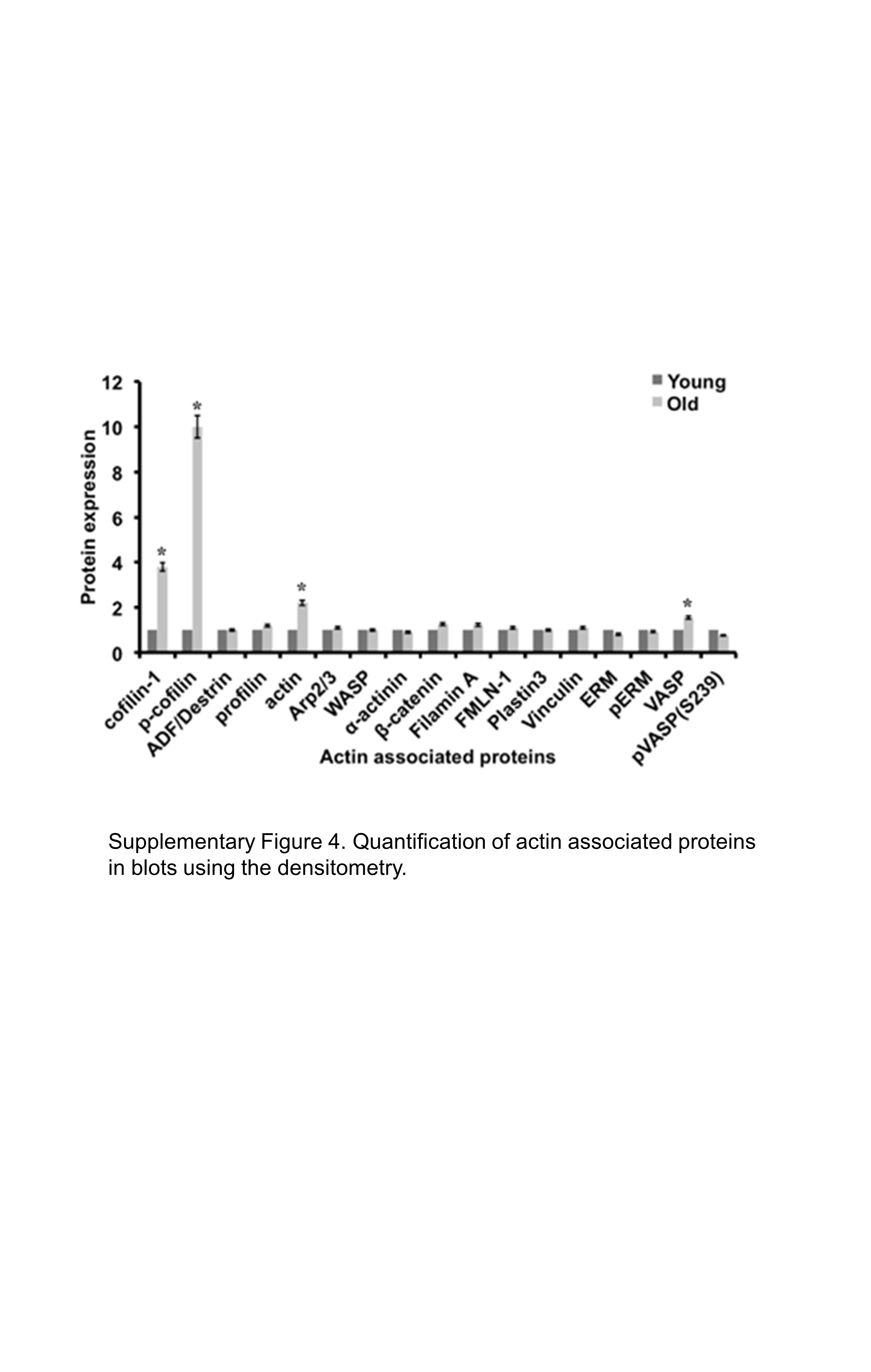

Supplement: Supplementary file 4 — Figure S4 [file ACEL-20-e13288-s004.TIF]

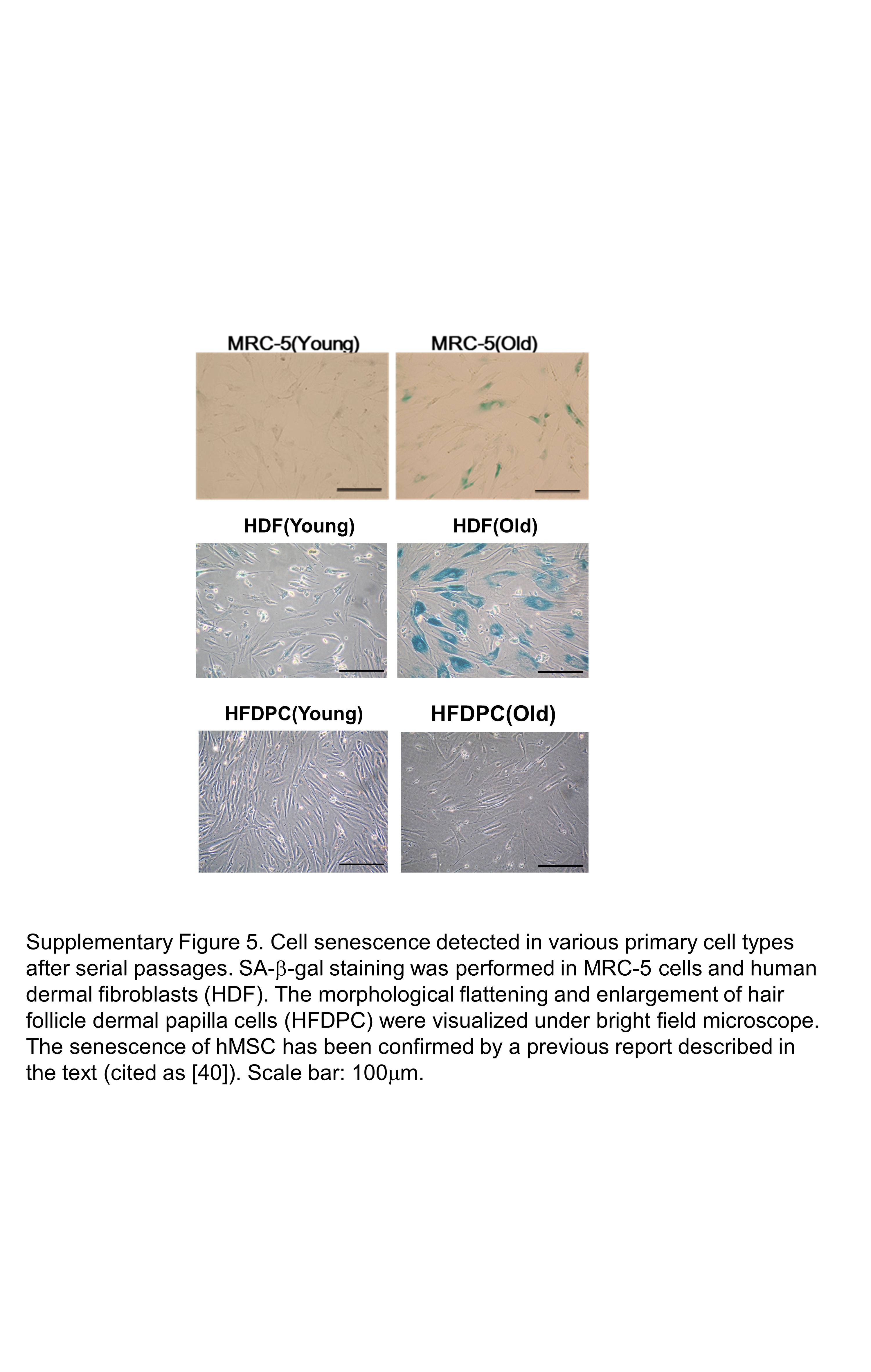

Supplement: Supplementary file 5 — Figure S5 [file ACEL-20-e13288-s005.TIF]

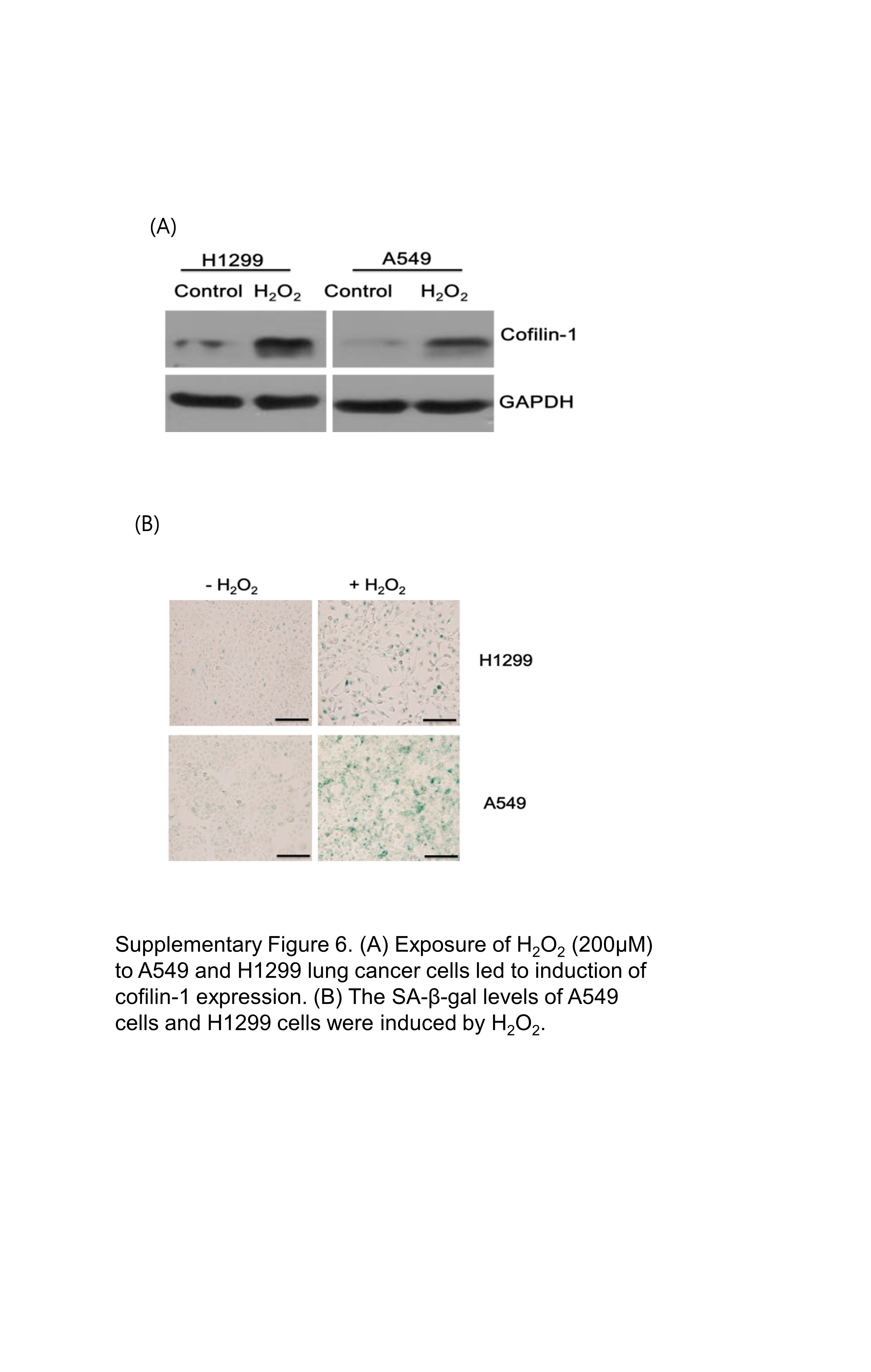

Supplement: Supplementary file 6 — Figure S6 [file ACEL-20-e13288-s006.TIF]

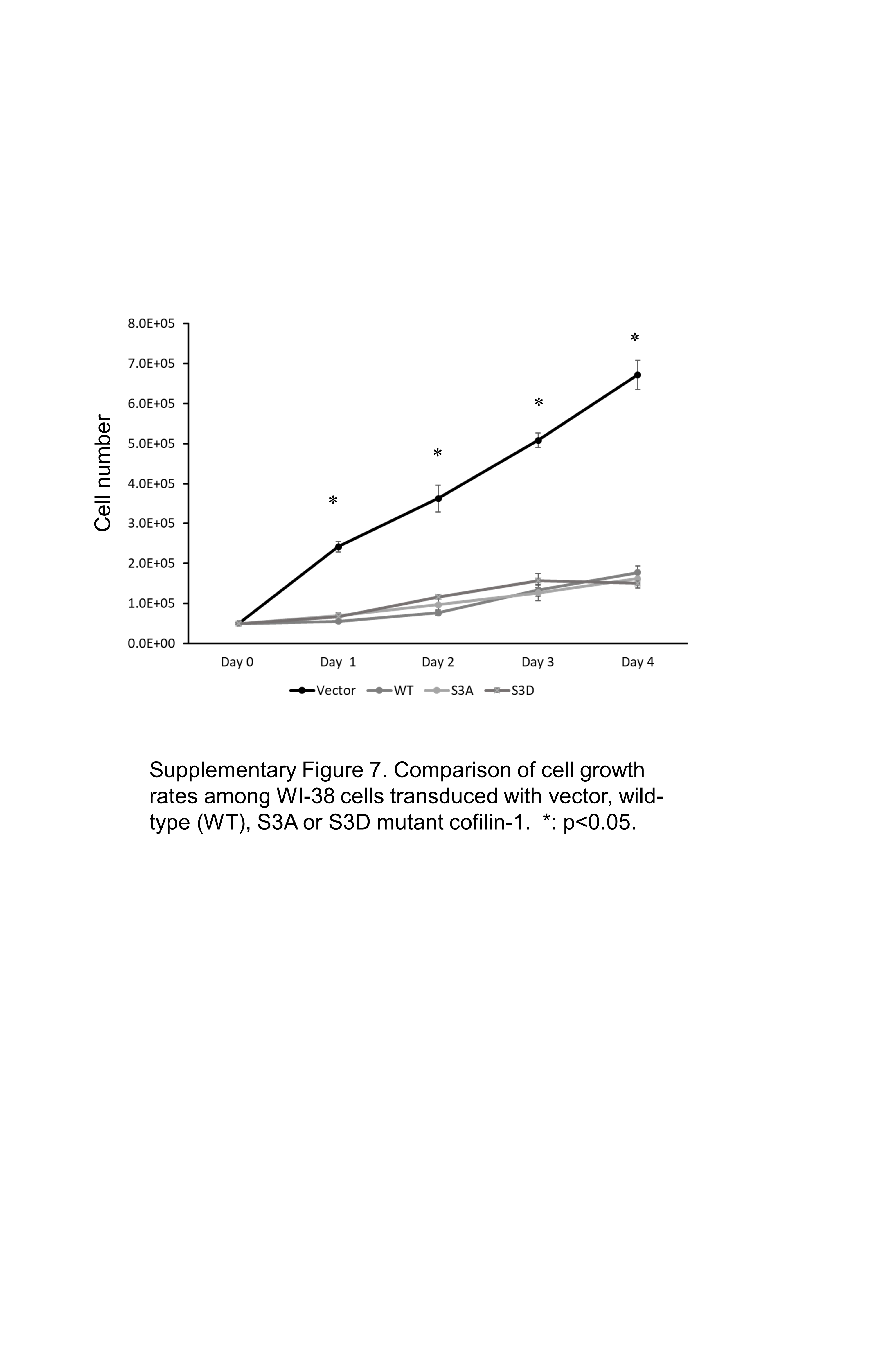

Supplement: Supplementary file 7 — Figure S7 [file ACEL-20-e13288-s007.TIF]

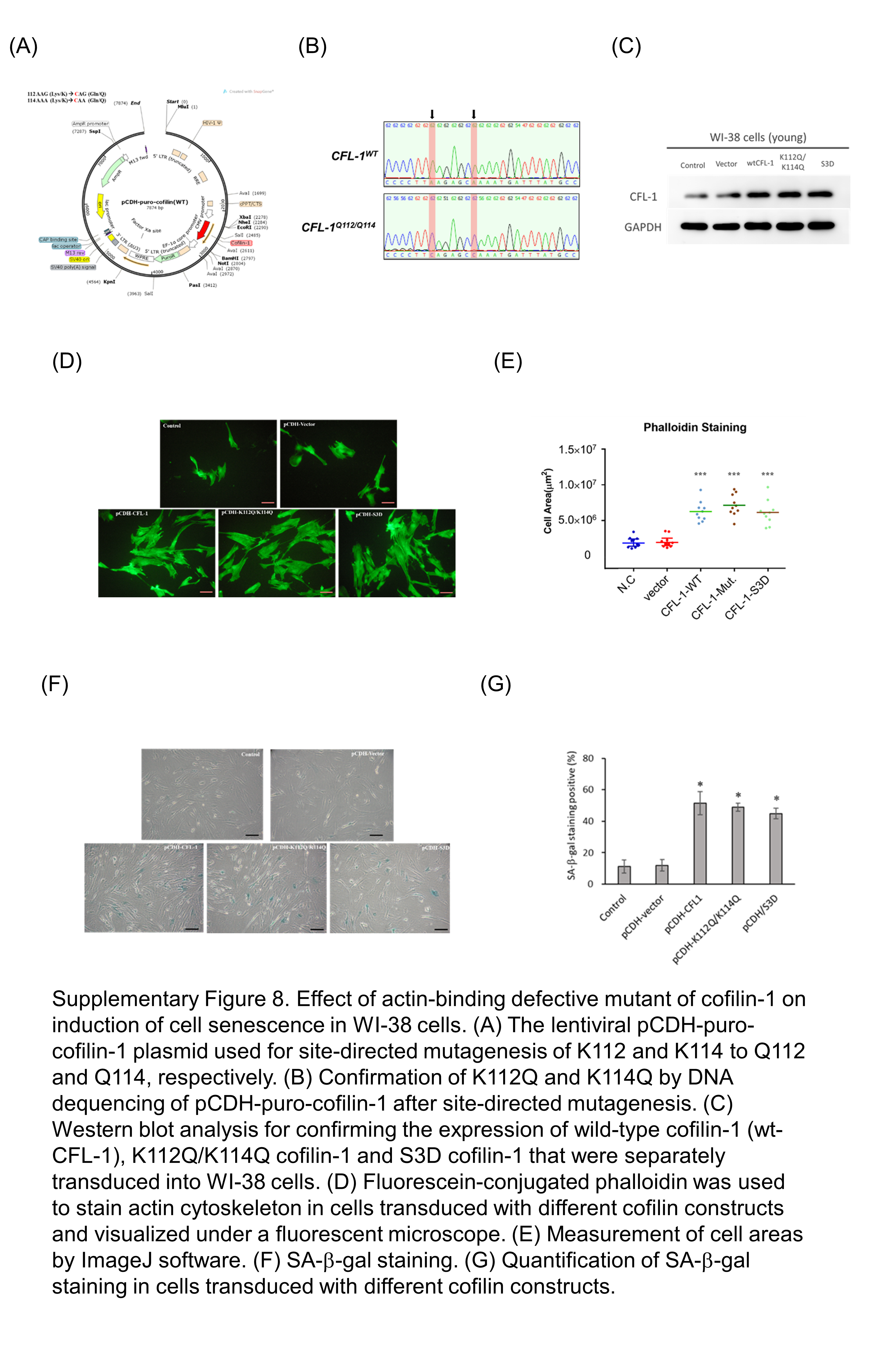

Supplement: Supplementary file 8 — Figure S8 [file ACEL-20-e13288-s008.TIF]

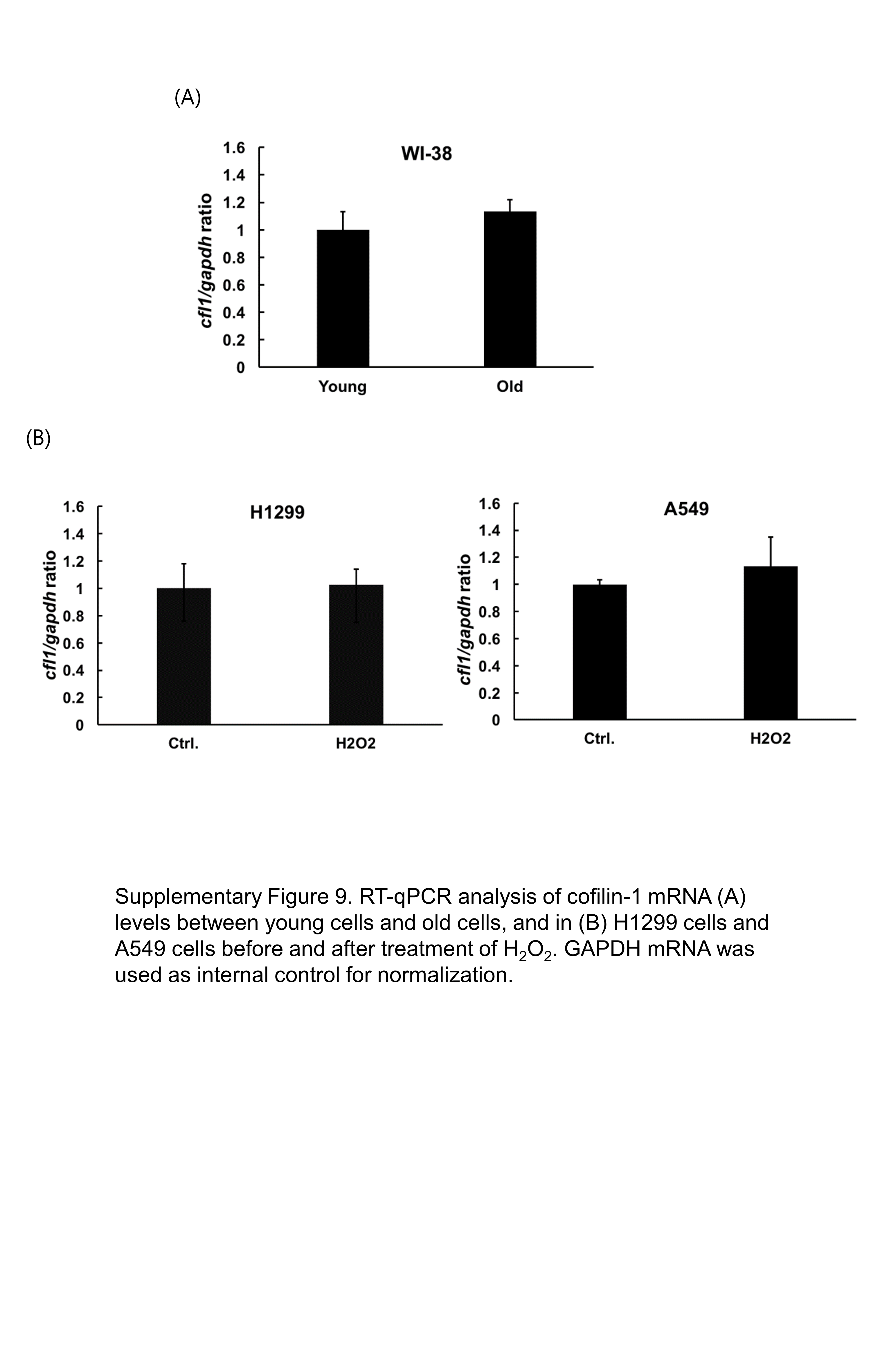

Supplement: Supplementary file 9 — Figure S9 [file ACEL-20-e13288-s009.TIF]

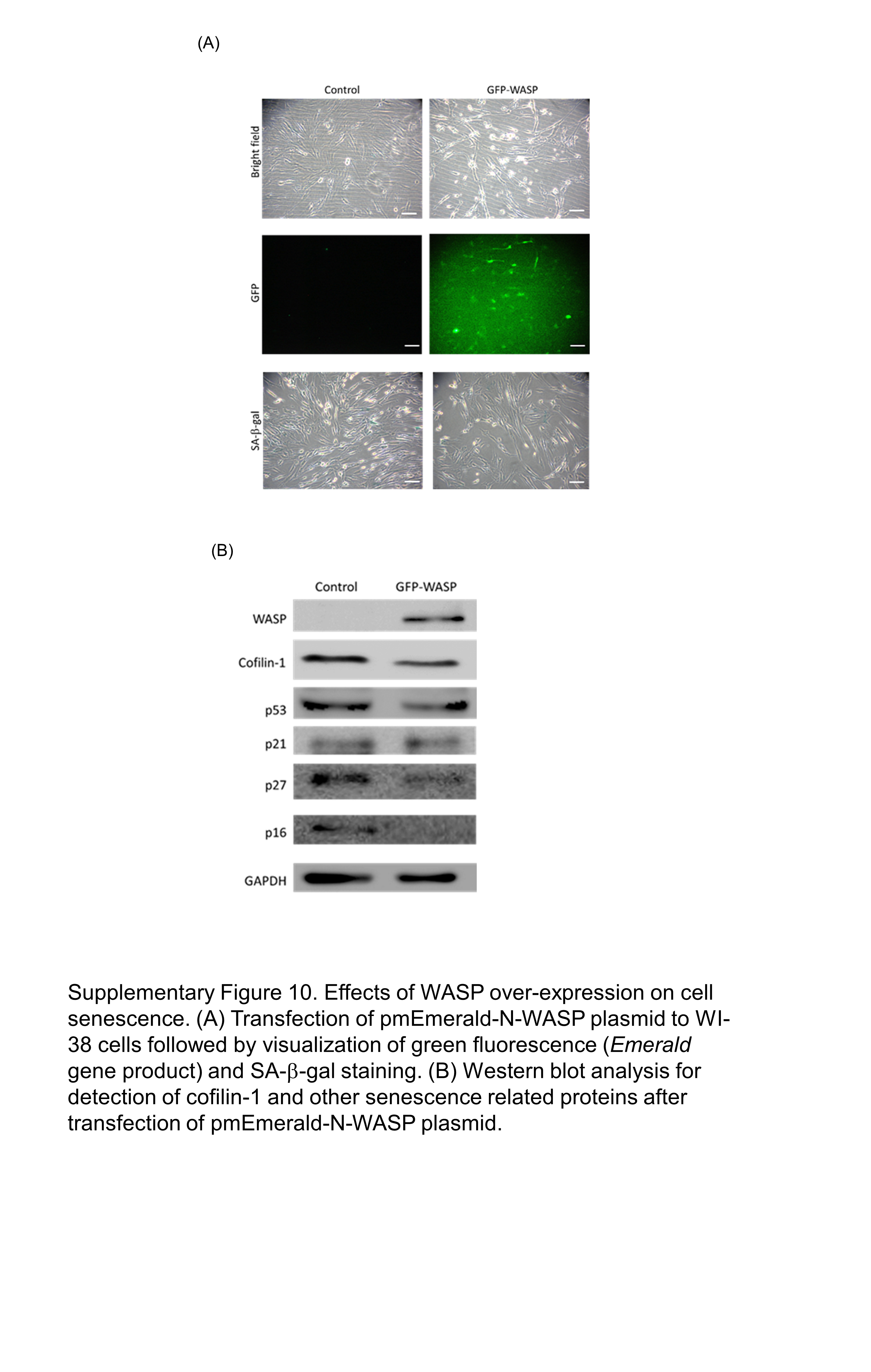

Supplement: Supplementary file 10 — Figure S10 [file ACEL-20-e13288-s010.TIF]

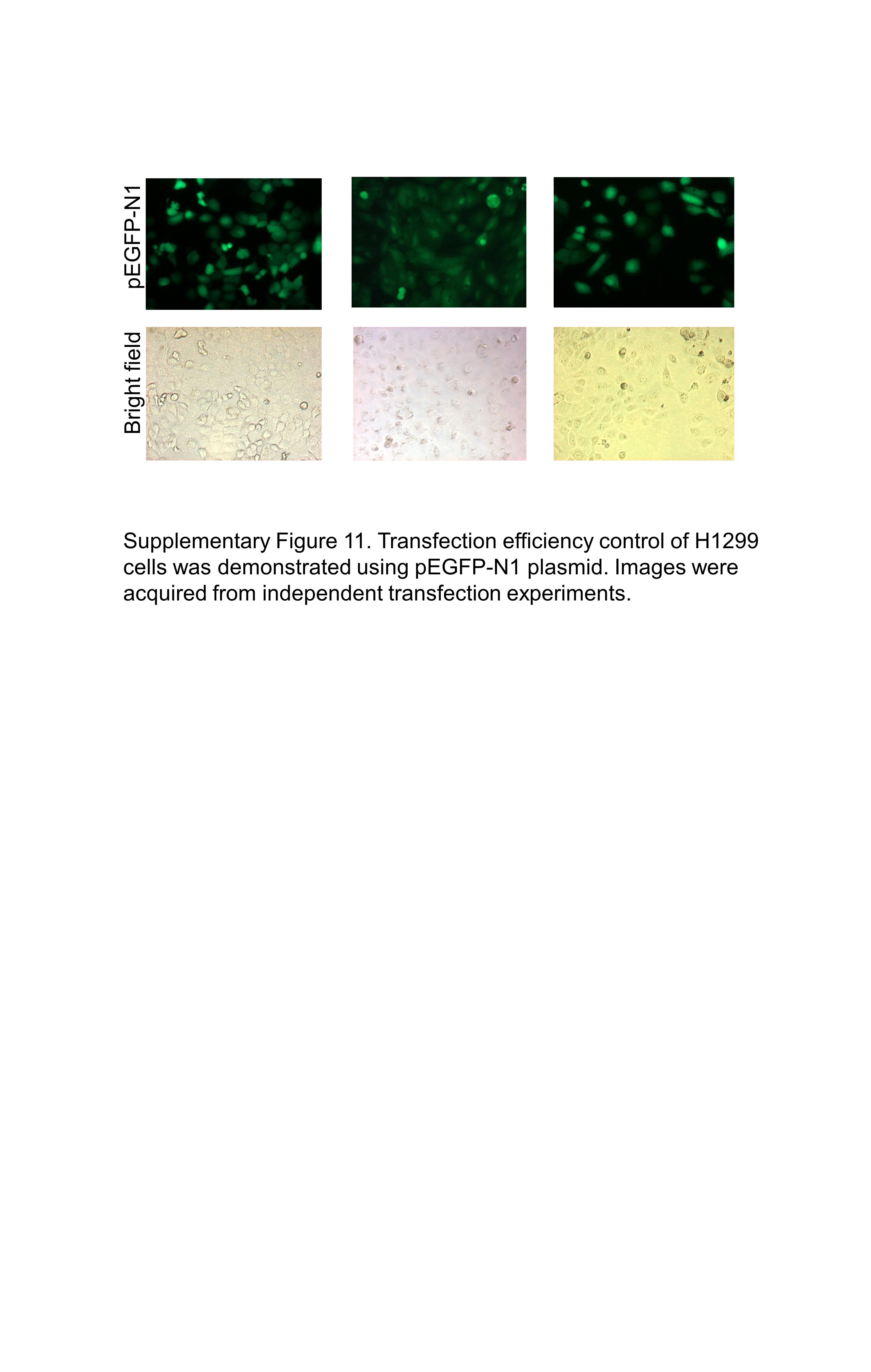

Supplement: Supplementary file 11 — Figure S11 [file ACEL-20-e13288-s011.TIF]

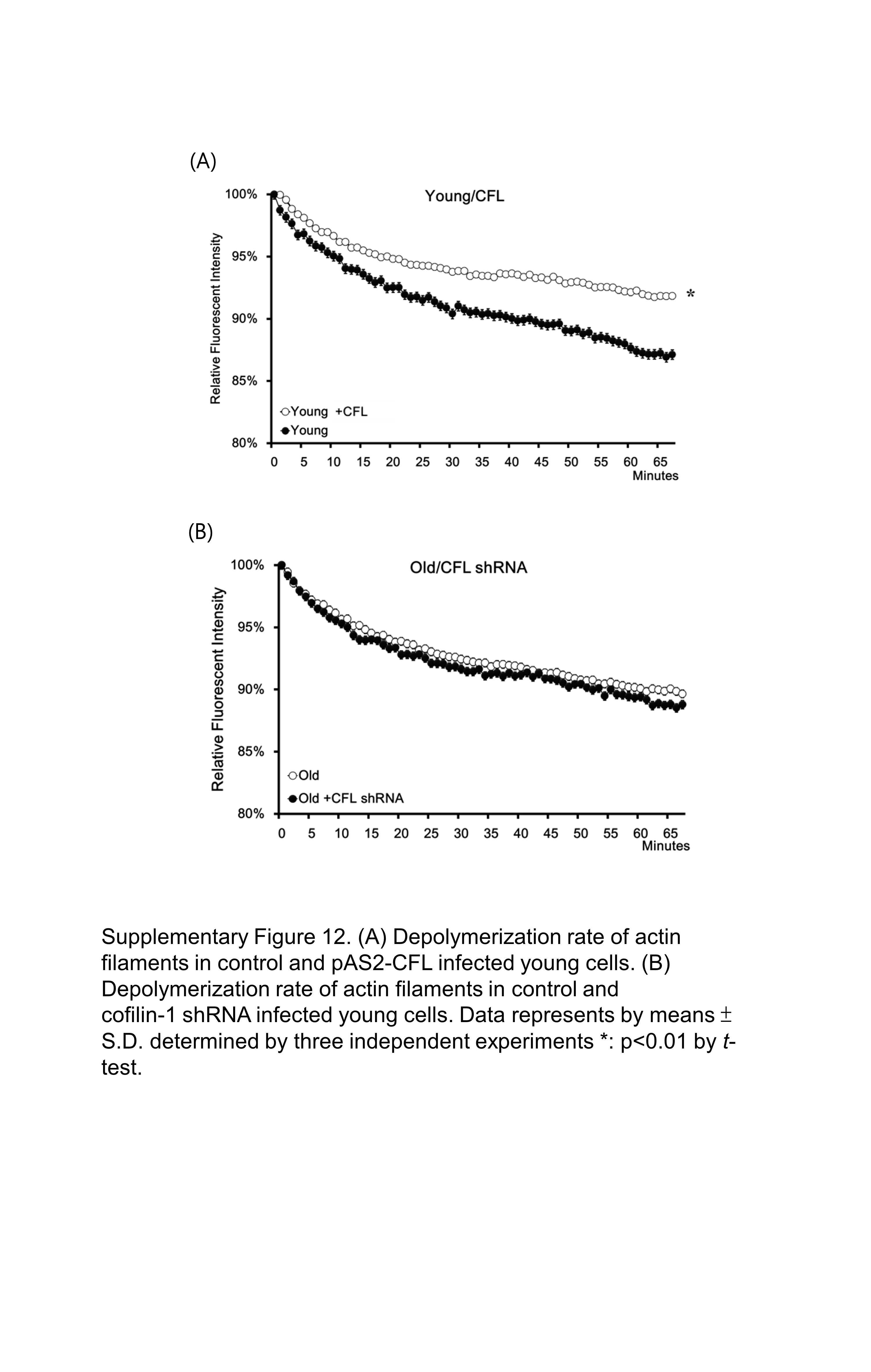

Supplement: Supplementary file 12 — Figure S12 [file ACEL-20-e13288-s012.TIF]

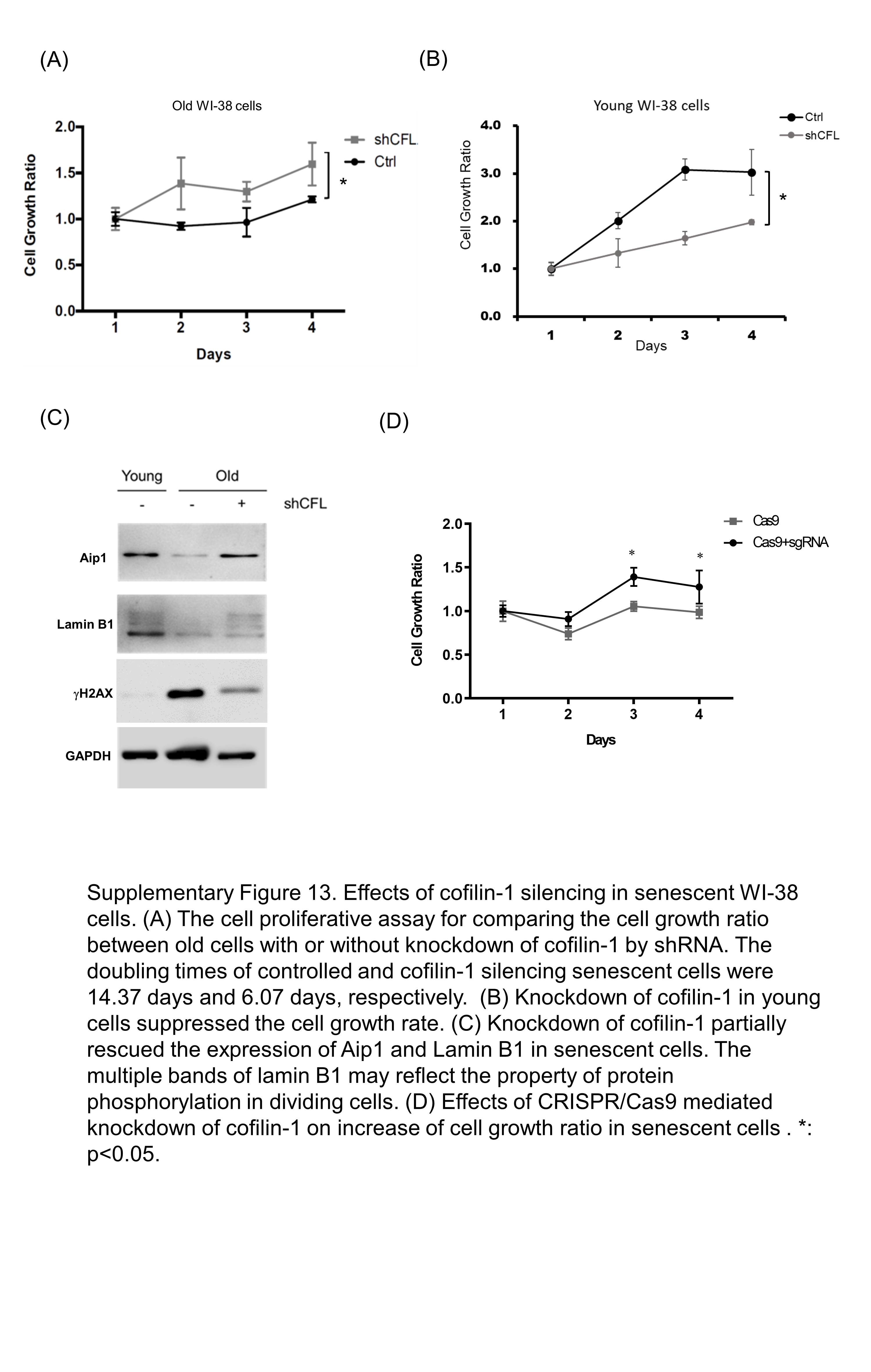

Supplement: Supplementary file 13 — Figure S13 [file ACEL-20-e13288-s013.TIF]

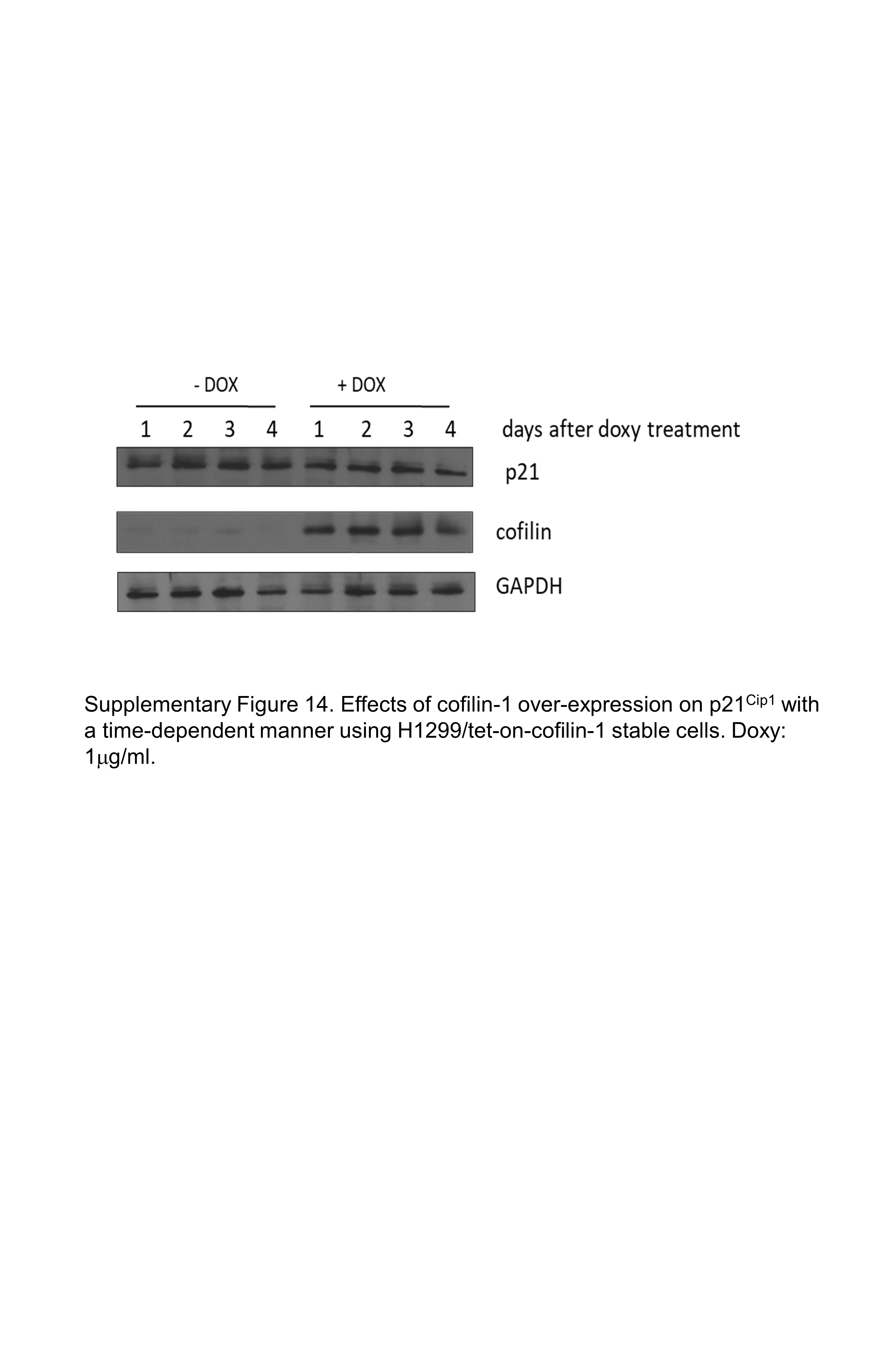

Supplement: Supplementary file 14 — Figure S14 [file ACEL-20-e13288-s014.TIF]

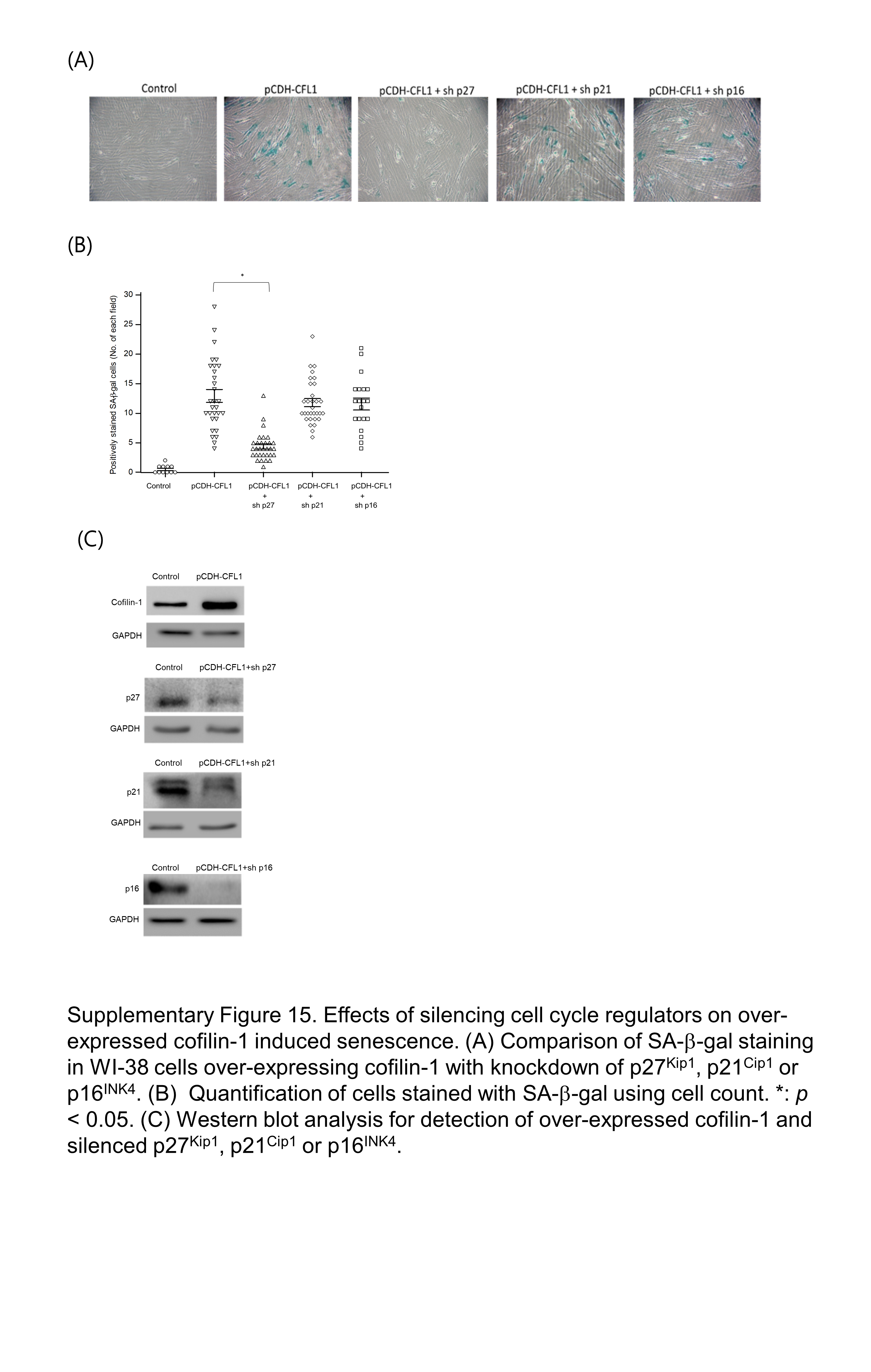

Supplement: Supplementary file 15 — Figure S15 [file ACEL-20-e13288-s015.TIF]

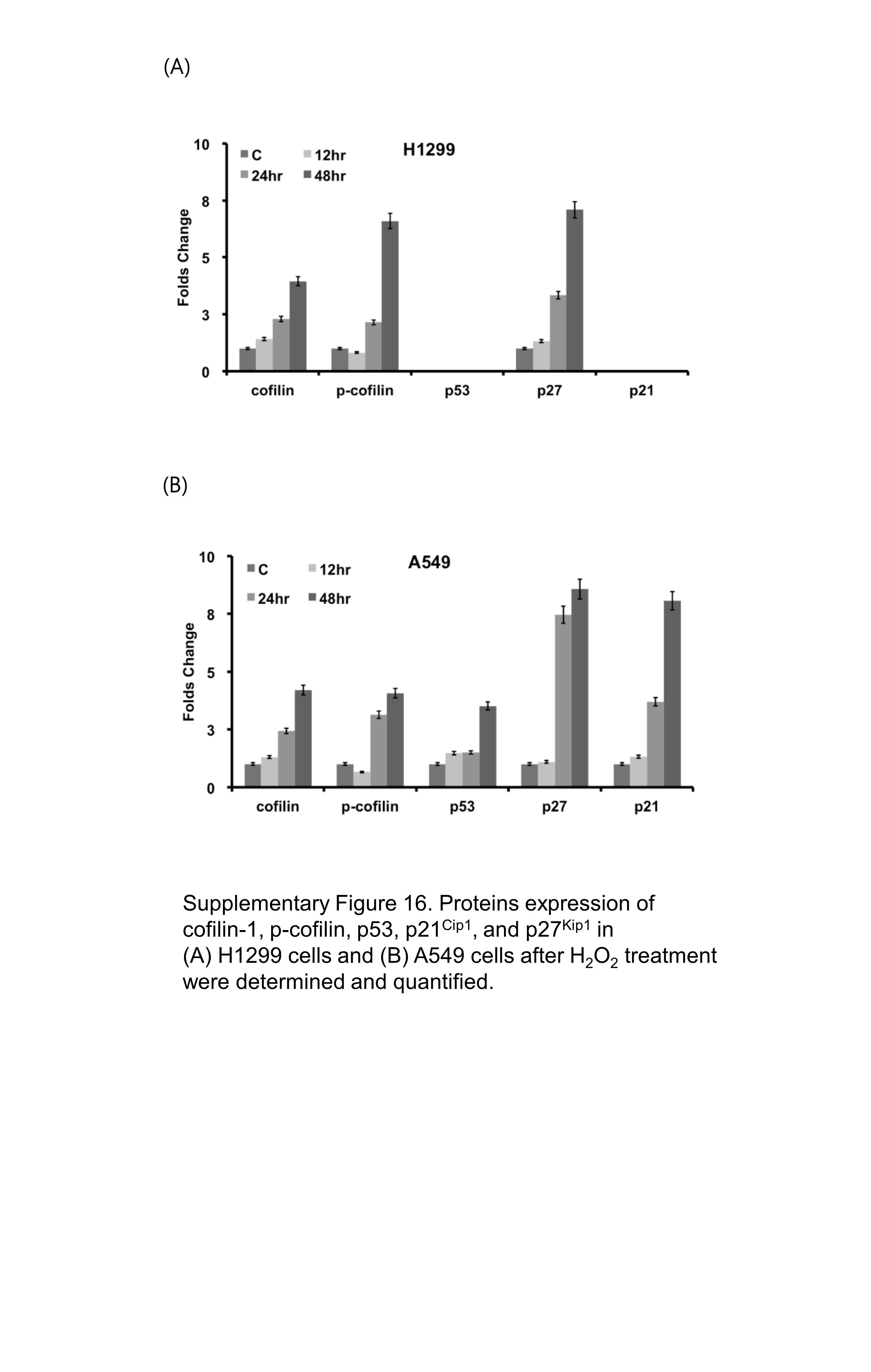

Supplement: Supplementary file 16 — Figure S16 [file ACEL-20-e13288-s016.TIF]

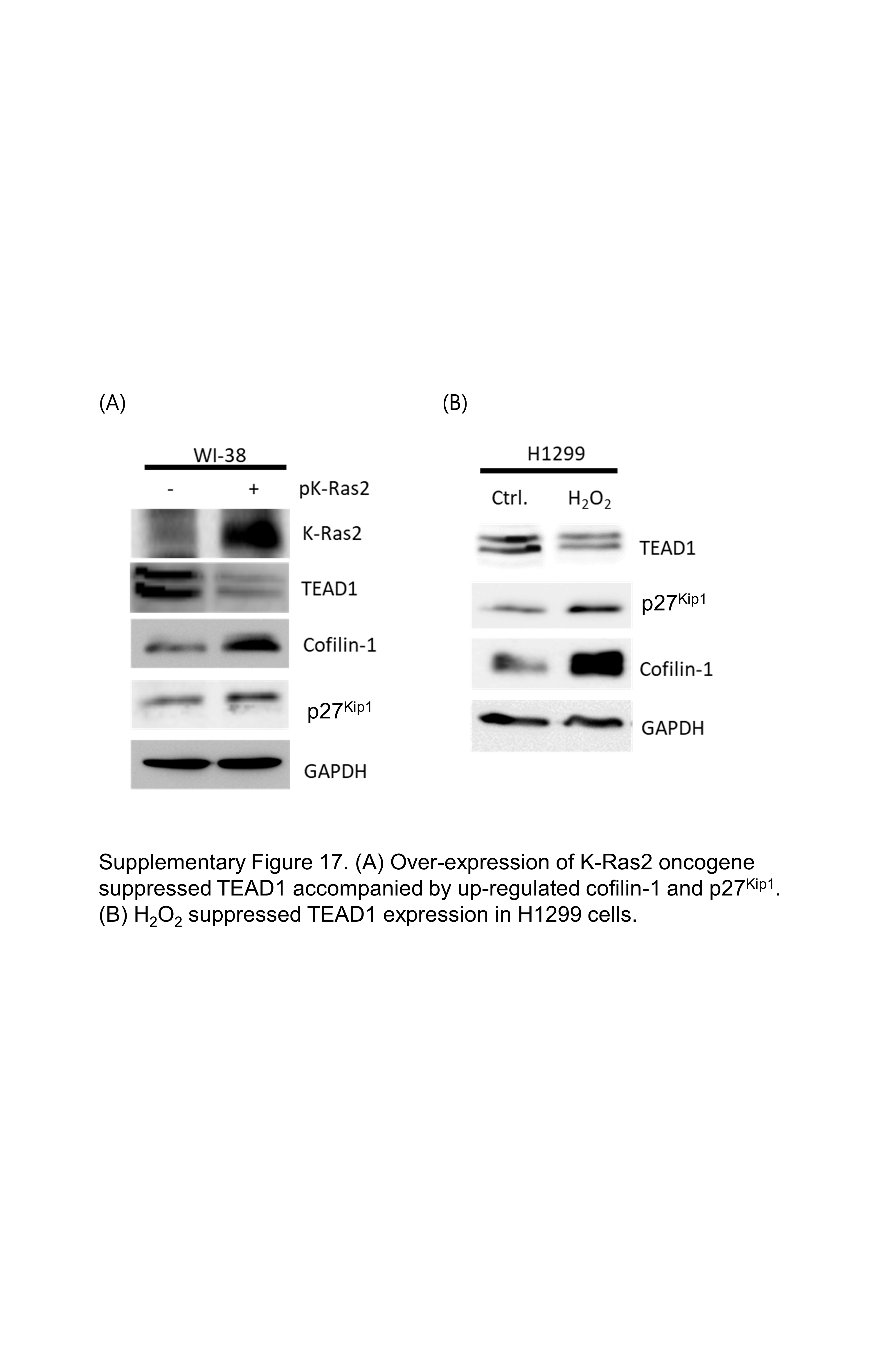

Supplement: Supplementary file 17 — Figure S17 [file ACEL-20-e13288-s017.TIF]

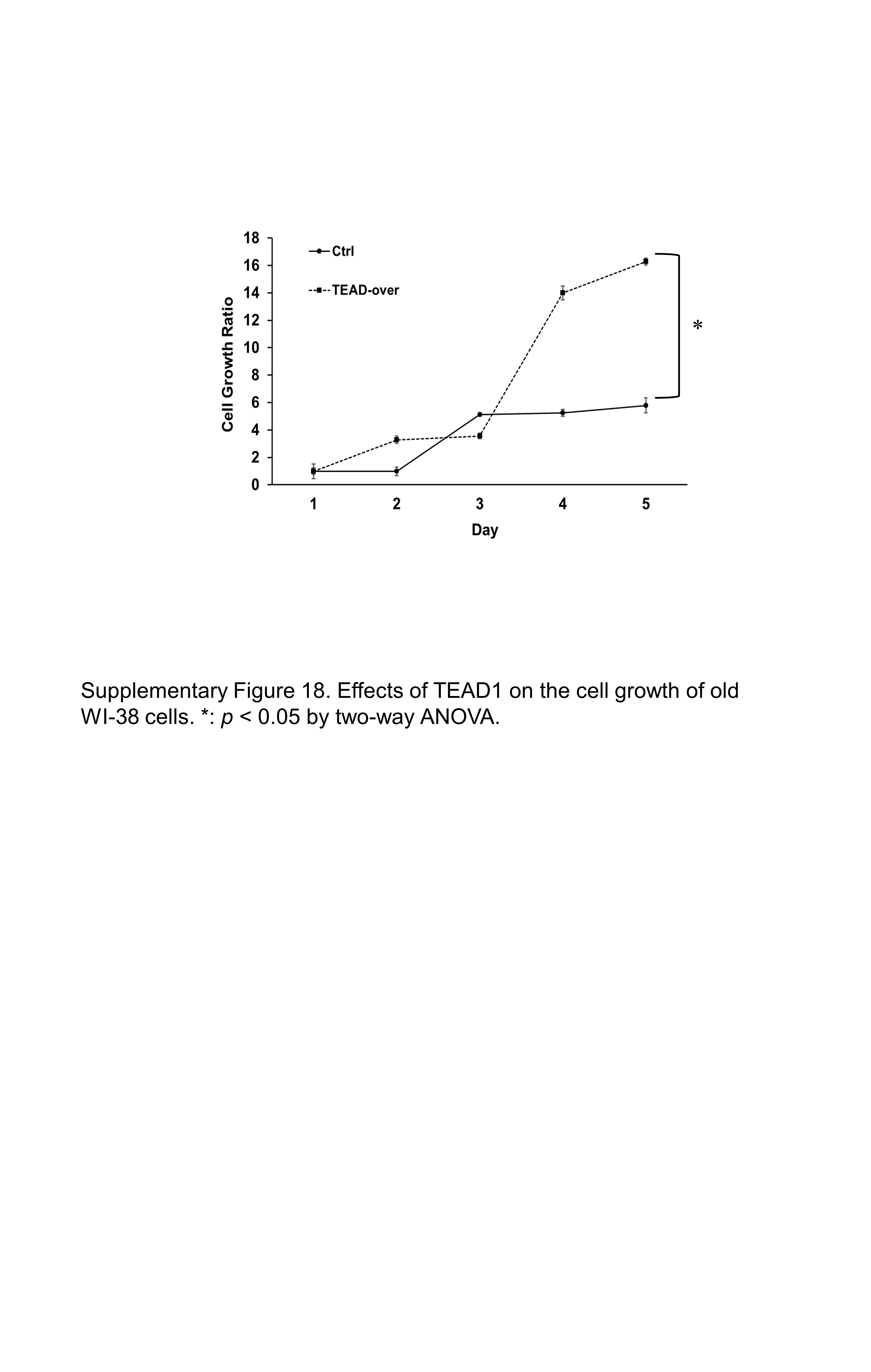

Supplement: Supplementary file 18 — Figure S18 [file ACEL-20-e13288-s018.TIF]

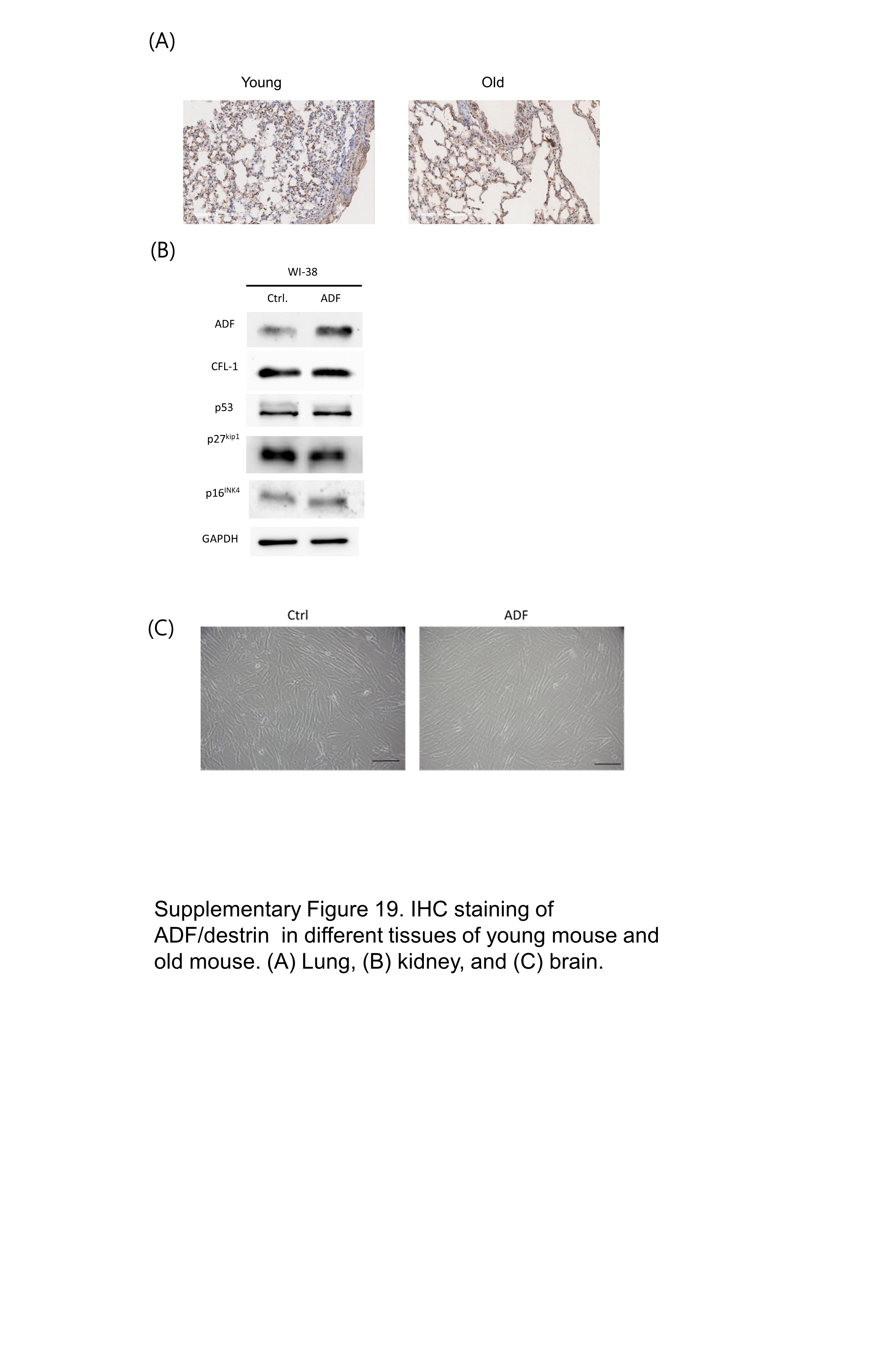

Supplement: Supplementary file 19 — Figure S19 [file ACEL-20-e13288-s019.TIF]

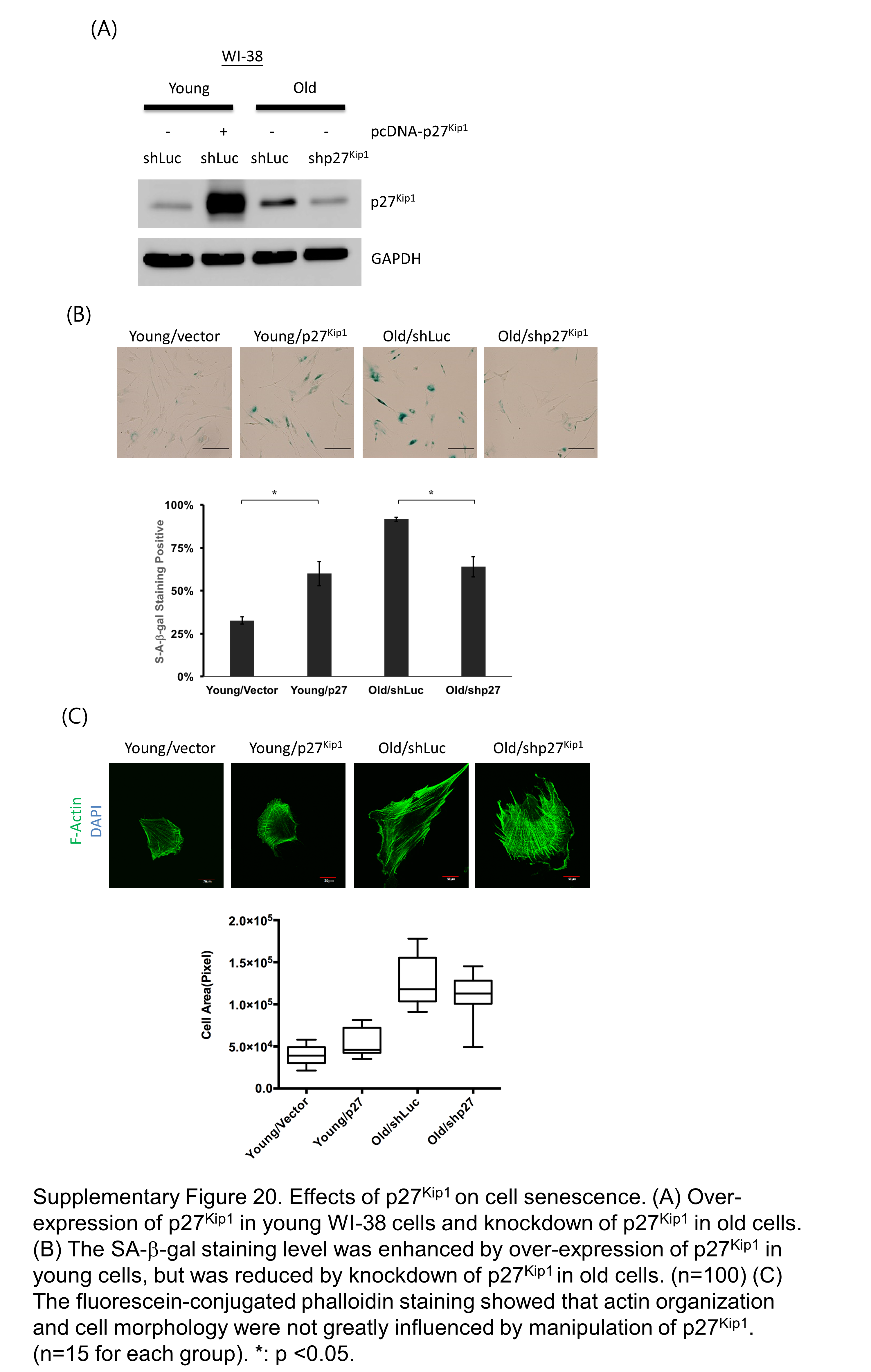

Supplement: Supplementary file 20 — Figure S20 [file ACEL-20-e13288-s020.TIF]
