# Supplementary material for: Up‐regulation of cofilin‐1 in cell senescence associates with morphological change and p27kip1‐mediated growth delay
Source: Aging Cell. 2020 Dec 18;20(1):e13288. doi: 10.1111/acel.13288 (PMC7811848; doi:10.1111/acel.13288)
Supplement: Supplementary file 21 — Table S1 [file ACEL-20-e13288-s021.ppt]

## Slide 1
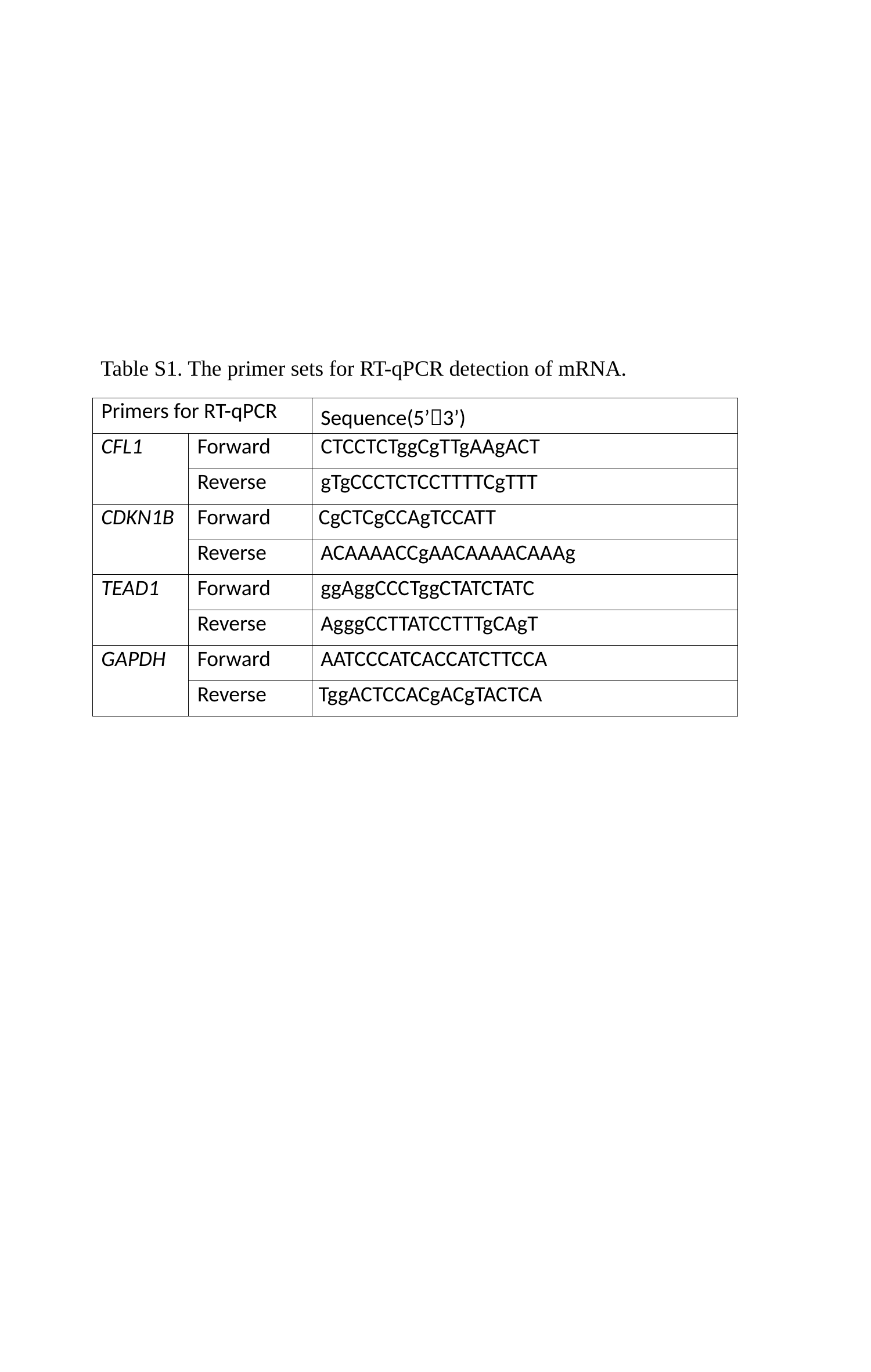

Table S1. The primer sets for RT-qPCR detection of mRNA.
| Primers for RT-qPCR | | Sequence(5’3’) |
| --- | --- | --- |
| CFL1 | Forward | CTCCTCTggCgTTgAAgACT |
| | Reverse | gTgCCCTCTCCTTTTCgTTT |
| CDKN1B | Forward | CgCTCgCCAgTCCATT |
| | Reverse | ACAAAACCgAACAAAACAAAg |
| TEAD1 | Forward | ggAggCCCTggCTATCTATC |
| | Reverse | AgggCCTTATCCTTTgCAgT |
| GAPDH | Forward | AATCCCATCACCATCTTCCA |
| | Reverse | TggACTCCACgACgTACTCA |
