# Supplementary material for: Up‐regulation of cofilin‐1 in cell senescence associates with morphological change and p27kip1‐mediated growth delay
Source: Aging Cell. 2020 Dec 18;20(1):e13288. doi: 10.1111/acel.13288 (PMC7811848; doi:10.1111/acel.13288)
Supplement: Supplementary file 22 — Table S2 [file ACEL-20-e13288-s022.ppt]

## Slide 1
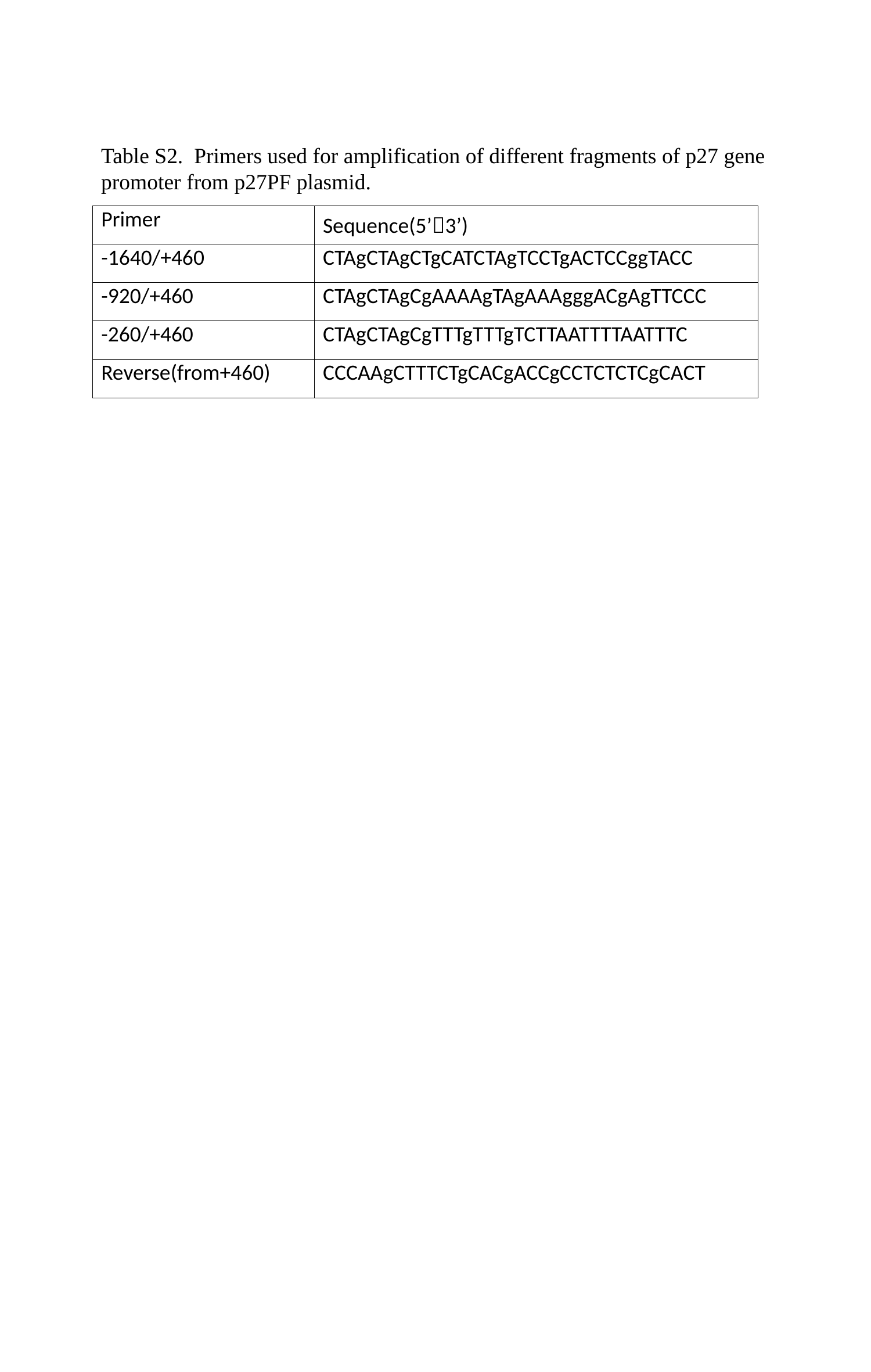

Table S2. Primers used for amplification of different fragments of p27 gene promoter from p27PF plasmid.
| Primer | Sequence(5’3’) |
| --- | --- |
| -1640/+460 | CTAgCTAgCTgCATCTAgTCCTgACTCCggTACC |
| -920/+460 | CTAgCTAgCgAAAAgTAgAAAgggACgAgTTCCC |
| -260/+460 | CTAgCTAgCgTTTgTTTgTCTTAATTTTAATTTC |
| Reverse(from+460) | CCCAAgCTTTCTgCACgACCgCCTCTCTCgCACT |
