# Supplementary material for: Up‐regulation of cofilin‐1 in cell senescence associates with morphological change and p27kip1‐mediated growth delay
Source: Aging Cell. 2020 Dec 18;20(1):e13288. doi: 10.1111/acel.13288 (PMC7811848; doi:10.1111/acel.13288)
Supplement: Supplementary file 23 — Supporting information [file ACEL-20-e13288-s023.docx]

**Experimental Procedures**

**Cell lines.** Human diploid fibroblast WI-38 cells were cultured in Minimum Essential Medium (MEM). H1299 cells, HEK293 cells, 293T cells, HT-29 cells, MRC-5 cells and A549 cells were cultured in Dulbecco's Modified Eagle's Medium (DMEM). Glioblastoma (GBM) S1R1 cells and H292 cells were cultured in RPMI 1460 medium. The pH value of all media were adjusted to 7.4 and supplemented with 10% fetal bovine serum (FBS), 2mM L-glutamate, and 50U/ml penicillin (Sigma-Aldrich, St. Louis, MO, USA). Human dermal fibroblasts (HDF) and the culture medium were purchased from American Types of Culture Collection (ATCC, Manassas, VA, USA). The hair follicle dermal papilla cell (HFDPC) and the growth media were purchased from Cell Application, INC. (San Diego, CA, USA). H1299/*tet*-*on-*cofilin-1 cells were cultured as described previously (Tsai et al., 2015). All cell cultures were maintained in a 37°C, humidified incubator (5% CO_2_ and 95% air), and were passaged when they reached 80% confluence.

**Plasmids construction.** The shRNA plasmids pLKO.1-shCFL1, pLKO.1-shTEAD1, pLKO.1-shLuc, pLKO.1-shp27^Kip1^, pLKO.1-shp21^Cip1^, and pLKO.1-shp16^INK4^ were purchased from the RNAi core facility at Sinica Academy, Taipei, Taiwan. Cofilin-1 cDNA was amplified by reverse transcriptional-polymerase chain reaction (RT-PCR) and cloned into the pAAV-MCS vector (Agilent Technologies, Santa Clara, CA, USA) *via* EcoRI and BamHI sites. pLKO.1-shLuc was used as an off-target control for all gene targeting shRNA. The CMV promoter fused to cofilin-1 cDNA was then subcloned into the pLKO-AS2-puro plasmid *via* the MluI and BamHI sites (pAS2-CFL). These plasmids were used to produce lentiviral particles for cell infection. The cDNA of flag-cofilin-1 digested from pBIG2i-Cofilin-1 (Lee, Mazzatti, Yun, & Keng, 2005) was inserted into lentiviral vector pCDH-CMV-MCS-EF1-puro (System bioscience, California, USA) *via* EcoRI and BamHI sites. The pGS-3HA-TEAD1, pBabe-KRas-WT, and pCMV5-human-p27 plasmids were purchased from Addgene (Addgene, Cambridge, MA, USA). TEAD1 cDNA was then PCR amplified using primers flanked with NheI and EcoRI sites. KRas cDNA and p27^Kip1^ cDNA were PCR amplified using primers flanked with NheI and BamHI sites. ADF cDNA was amplified from WI-38 cells using RT-PCR, and cloned to a TA cloning kit. TEAD1, KRas, p27^Kip1^ and ADF cDNA were then separately subcloned into pCDH-CMV-MCS-EF1-puro plasmid. The p27PF reporter plasmid harboring 3,556bp length of p27^Kip1^ gene promoter was a gift from Dr. Toshiyuki Sakai (Minami, Ohtani-Fujita, Igata, Tamaki, & Sakai, 1997). This reporter plasmid was used as a template to construct serial deletions of p27^Kip1^ gene promoter using high fidelity KAPA HiFi PCR kit (Kapa Biosystems, Inc., Woburn, MA, USA). Different primer sets were flanked with NheI and Hind III sites on 5’ end and 3’ end for amplification of deleted fragments of p27^Kip1^ gene promoter, respectively (Table S1). Full length promoter and all PCR products were separately digested and subcloned into the pGL4.1-Luc2 vector (Promega Corporation, Madison, WI, USA) using the T4 DNA ligase (Cell Signaling Technology, Danvers, MA, USA). The Y68F mutant cofilin-1 construct (pHan-cof-Y68F) was a generous gift received from Dr. J. L Guan (Yoo, Ho, Wang, & Guan, 2010). A wild-type cofilin-1 cDNA was PCR amplified using a forward primer 5’-CCGGGATCCATGGCCTCCGGTGTGG-3’ and a reversal primer 5’-CCGGAATTCCTCACAAAGGCTTGCCC-3’ followed by BamHI and EcoRI digestion and subcloned to the pHan vector. The pmEmerald-N-WASP plasmid was a generous gift offered by Dr. Jean-Cheng Kuo in Institute of Biochemistry and Molecular Biology, National Yang-Ming University.

**Determination of population doubling level (PDL).** WI-38 cells purchased from ATCC was PDL at 18 and the number of harvested cells were approximately 4 times more than initial seeding number every passage (Houghton & Stidworthy, 1979). PDL= 3.32 x log (Xe/Xb)+S, where Xb is the cell number at the beginning of the incubation time; Xe is the cell number at the end of the incubation time; S is the PDL of starting time point.

**Senescence-associated-β-Galactosidase (SA-β-gal) staining.** Cells were fixed using 0.2% glutaraldehyde plus 2% formaldehyde at 37°C for 5 min, and then rinsed with phosphate buffered saline (PBS). The staining solution (1 mg/ml of 5-Bromo-4-chloro-3-indolyl β-D-galactopyranoside, 5mM potassium ferricyanide, 5mM potassium ferrocyanide, 150mM NaCl, 1mM MgCl_2_, 40mM citric acid (pH 6.0)) was added into the culture dishes and incubated at 37°C for 24 hours. For staining of animal tissues, 5μm frozen tissue sections were fixed with 0.5% glutaraldehyde and stained as described above. The stained cells were visualized under an optical microscope with digital camera (Olympus, Center Valley, PA, USA). Quantification was determined by counting the number of positive stained cells per 100 randomly selected cells.

**Telomere length assay.** Measurements of telomere length (TL) including designs of standard, primer sets, and PCR condition were followed by a previous report (O'Callaghan, Dhillon, Thomas, & Fenech, 2008). Briefly, the Cawthon’s quantitative real-time polymerase chain reaction (qRT-PCR) assay was used to obtain the absolute TL value. A 84bp oligomer standard with 14 times TTAGGG repeats was used to draw a standard curve by Ct number against log[TL(kb)] generated by serial dilutions of standard. Genomic DNA was extracted using a DNA extraction kit according to the manufacturer’s instruction (Geneaid Biotech Ltd., New Taipei, Taiwan). The quality was determined by O.D.260/280. Genomic DNA (100ng) was subjected to qRT-PCR and the Ct number was fit to the standard curve to calculate the TL.

**Immunofluorescence staining and confocal microscopy.** Cells were seeded onto the cover slips for 48 hours and then fixed with 4% paraformaldehyde. Triton X-100 (0.1%) was then used for permeabilization of cell membrane. After blocking in 10% FBS, cells were subsequently incubated with specific primary antibody followed by Alexa-fluor-conjugated secondary antibody (ThermoFisher Scientific, Waltham, MA, USA) in 37°C. For nuclear staining, 4',6'-diamidino-2-phenylindole (DAPI) was used before seal. The primary antibodies used for indirect immunofluorescence staining included anti-cofilin-1 antibody (Genetex Inc., Irvine, CA, USA), and anti-Ki-67 antibody (Millipore, Billerica, MA, USA). Fluorescein-conjugated phalloidin (ThermoFisher Scientific, Waltham, MA, USA) was used for fluorescent staining of actin filaments. Images were acquired using a confocal fluorescence microscope with cooled CCD camera (Leica TCS SP2, Leica Microsystems Ltd., Buffalo Grove, IL, USA). For tissue sections, the samples were prepared as described in IHC staining, and the staining procedure for actin cytoskeleton was the same as above (N=3).

**Western blot analysis and antibodies.** The immunoblotting experiments were conducted according to a previous report (Tsai et al., 2009). The cell lystes of hMSC were generously provided by Dr. Shih-Chieh Hung (Hung et al., 2002). The primary antibodies used were: anti-cofilin, anti-p27^Kip1^, anti-profilin, anti-Arp2/3, anti-Sp1, anti-ADF/Destrin, anti-AP2, anti-CTF, anti-p16^INK4^, anti-GAPDH, anti-α-actinin, anti-β-catenin, anti-filamin A, anti-FMNL1, anti-plastin 3, anti-vinculin, anti-WASP, anti-KRas, anti-lamin B1, anti-HAtag, anti-Histag and anti-p53 (Genetex Inc., Irvine, CA, USA); anti-phospho-specific (ser3) cofilin (Santa Cruz Biotechnology Inc., Dallas, TX, USA); anti-p21^Cip1^ (Millipore, Billerica, MA, USA); anti-TEAD1, anti-TEAD4, anti-ubiquitin, and anti-γH2AX (Cell Signaling Technology, Danvers, MA, USA), and anti-actin (Sigma-Aldrich, St. Louis, MO, USA); anti-Aip1 (Abcam, Cambridge, MA, USA). The actin re-organization antibody sampler kit (Cell Signaling Technology, Danvers, MA, USA), including anti-VASP and anti-ERM antibodies was also used for Western blot analysis. The secondary antibodies were anti-mouse and anti-rabbit IgG1 antibodies (Millipore, Billerica, MA, USA). The secondary anti-rabbit antibody recognizing native primary antibody only was purchased from Sigma (Sigma-Aldrich, St. Louis, MO, USA).

**Cell cycle analysis.** Cell cycle was determined by flow cytometry as reported before (Tsai et al., 2009).

**Cell growth assay.** Cells (5x10^4^) were cultured in 6cm dishes and incubated in a humid incubator at 37°C as mentioned above. After 48 hours of incubation, cells were trypsinized and counted every 24 hours until the end of analysis using hemocytometry. Triplicate experiments were performed for each time point.

**Determination of cell morphology and size.** The “Cell Morphology Analyzer” programmed by the Matlab software (Mathoworks®, Natick, MA USA) was used to quantify the cell shapes. Briefly, cell images stained by fluorescein-conjugated phalloidin were loaded into the cell morphology analyzer. The contour of each cell was firstly defined using a wand tool in the software, and a smooth contour of each cell was drawn by the cell morphology analyzer accordingly (Supplementary Figure 2). The pixel numbers were recorded by different color intensity between fluorescent cells and black background. The source code of Cell Morphology Analyzer will be provided upon request.

**Fluorescent pyrene-conjugated actin polymerization assay.** The actin polymerization and de-polymerization rates were measured using an actin polymerization Biochem Kit™ (Cytoskeleton Inc., Denver, CO, USA). The procedures were followed by the manufacturer’s instructions. In brief, the actin polymerization was measured by incubating adenosine-5'-triphosphate (ATP) supplemented pyrene G-actin mixture with cell lysate harvested in cell lysis buffer at pH=6.8. For actin de-polymerization rate, pyrene G-actin was added ATP supplemented Tris-HCl buffer for 1 hour incubation in room temperature to allow pyrene F-actin formation and then mixed with cell lysate (pH=8). The fluorescent signals in both assays were acquired by the Infinite M2000 PRO TECAN reader (Tecan Group Ltd., Männedorf, Switzerland) every 5 minutes till the end of indicated time point.

**Two-dimensional gel immunoblotting analysis.** The protocol of 2D gel blotting analysis was referred to a previous report with modifications (Tahtamouni, Shaw, Hasan, Yasin, & Bamburg, 2013). In brief, 180μg protein lysate was run on a 2D gel. Each sample was analyzed by 13cm, 2-DE linear immobilized pH gradient (IPG) Dry Strip (Immobiline^TM^ Dry Strip pH 3-10 NL, 13cm, GE healthcare, Waukesha, WI, USA) with pH 3–10. The strip was immersed in 400ml buffer (7M urea, 2M thiourea, 1% DTT, 0.5% IPG buffer, 4% CHAPS). Total protein lysate were precipitated with chloroform/methanol and resolved with rehydration buffer. Isoelectric focusing (IEF) was followed by cup-loading protocols, i.e. 500 V, 5 h (Step-and-hold); 1000V, 1 h (Gradient); 8000V, 2 h (Gradient); 8000V, 10 h (Step-and-hold) on an IPGphor (Ettan IPGphor II, GE healthcare, Waukesha, WI, USA). The strips were sequentially re-equilibrated in equilibration buffer (1% dithiothreitol, 6 M urea, 30 % glycerol, 10% SDS, 75 mM Tris-HCl (pH8.8)) and resolved using 15% 2D-PAGE. The resolved proteins were then transferred to a nitrocellulose membrane, which was blocked with 4% skimmed milk for 1 hour, and then incubated with primary antibody at 4^o^C overnight followed by horseradish peroxidase-conjugated secondary antibodies. The primary antibodies were anti-cofilin-1 (1:1000), and phosphor-specific anti-cofilin-1 (1:750).

**Site-directed mutagenesis.** A kit was used to create an actin-binding defective (K112Q/K114Q) mutant cofilin-1 by following the manufacture’s protocol (Applied Biological Materials Inc., Richmond, BC, Canada). The sequences of forward primer: 5’-CTGCGCCCCTTCAGAGCCAAATGATTTATGC-3’; reversal primer: 5’- GCATAAATCATTTGGCTCTGAAGGGGCGCAG-3’ were used to amplify mutant cofilin-1 from pCDH-puro-cofilin construct.

**Immunohistochemistry (IHC) staining.** Tissues resected from young and old mice were rinsed with PBS and fixed in 4% paraformaldehyde with gentle shaking overnight at 4°C. Six-week mice were regarded young adults according to the mouse breeding guidelines of Johns Hopkins University (http://web.jhu.edu/animalcare/policies/index.html). For cryosections, the fixed samples were then embedded in OCT (optimal cutting temperature) solution and stored in -80°C. Tissue sections (5μm) were then subjected to the IHC staining protocols. For paraffin embedded sections, tissue sections were de-paraﬃnized in xylene (Sigma-Aldrich, St. Louis, MO, USA) for 30 minutes followed by rehydration in graded ethanol from 95%, 75% to 50% and finally in desterilized water or PBS. For antigen retrieval, tissues were boiled in 1mM EDTA Buffer (pH=8.0) for 20 minutes. The tissue sections were blocked in goat serum containing 5% H_2_O_2_, and then incubated with different primary antibodies at 37°C for 1.5 hours. The slides were then incubated with HRP-conjugated secondary antibodies (Sigma-Aldrich, St. Louis, MO, USA) at 37°C for 1 hour. Finally, the tissue sections were incubated with 3′,3′-diaminobenzidine (DAKO, Dako Denmark A/S Produktionsvej 42 DK-2600 Glostrup Denmark) until a brown color was developed and then counterstained with hematoxylin. For H&E staining, tissue sections were stained by hematoxylin and eosin after antigen retrieval. The images of tissue sections were acquired using an optical microscope with digital camera (Olympus, Center Valley, PA, USA). Arbitrary scoring of IHC was set up from 0-4: score 0 (negative), score 1, low intensity and less than 24% distribution; score 2, low to moderate intensity and 25%-49% distribution; score 3, moderate to high intensity and 50%-74% distribution; score 4, high intensity and more than 75% distribution. The percentage distribution was the positive stained cells relative to total area.

**Lentiviral-mediated gene transduction.** Human embryonic kidney 293T cells (2.5 × 10^6^ cells) were cultured to reach about 70% confluence. Cells were then co-transfected with lentiviral plasmids and the viral packaging plasmids CMV-△R8.91 and pMDG using the jetPEI transfection reagent (Polyplus-transfection SA, Illkirch, France). After 16 hours of transfection, the supernatant was removed and replaced with fresh medium containing 1% bovine serum albumin (BSA). The medium containing virus particles were collected after 36 hours of incubation. The viral soup was then ultra-centrifuged at 110,000 × g for 2 hours, and pellets were resuspended in serum-free medium. For cell infection, the viral soup (multiplicity of infection, M.O.I=2) was mixed with 8 μg/ml of polybrene (Sigma-Aldrich, St. Louis, MO, USA) and added to the target cells for 24 hours. The virus-containing medium were then replaced with fresh medium for an another 24–72hr prior to analysis.

**The CRISPR/Cas9 mediated gene knockdown**. The CRISPR/Cas-9 genetic editing system is to manipulate gene expression at the genomic level by disrupting the gene structure (Campenhout et al., 2019). The plasmids used for the CRISPR/Cas9 system were purchased from the RNAi core facility at Sinica Academy, Taipei, Taiwan. Briefly, p5w-Cas9.pBsd was derived from pLAS5w.pBsd lentiviral vector and the expression of Cas9 gene was driven by a human elongation factor 1α (EF1α) promoter. The pU6-sgRNA.pPuro lentiviral vector was inserted with the small guide RNA (sgRNA) pairs: 5’sgRNA (5’-GGTAGGGGTCGTCGACAGTC-3’) and 3’sgRNA (5’-GACTGTCGACGACCCCTACC-3’) to specifically target cofilin-1 gene in the genomes. Plasmids were transduced into cells by lentiviral infection as described above. After infection for 48 hours, cells were then treated with puromycin (1μg/ml) for 1 week to select out the uninfected cells, and collect the survived cells for Western blot analysis to verify the effect of cofilin-1 knockdown by CRISPR/Cas9 system to ensure the gene editing worked as expected.

**Quantification of gene transcription.** Quantitative reverse transcription – polymerase chain reaction (qRT- PCR) was performed to measure mRNA levels, In brief, total RNA was extracted from cells using the Trizol reagent (Life-Technologies Co, Grand Island, NY, USA) and quantified using the NanoDrop ND-1000 (Thermo Fisher Scientific, Waltham, MA, USA). The cDNA was generated from 2μg total RNA using SuperScript III reverse transcriptase (Thermo Fisher Scientific, Waltham, MA, USA). The cDNA products were then mixed with the Fast SYBR Green Master Mix (Applied Biosystems, Life-Technologies Co, Grand Island, NY, USA) and amplified in the StepOne Plus Real-Time PCR System (Applied Biosystems, Life-Technologies Co, Grand Island, NY, USA) according to the manufacturer’s instructions. Each datum was the mean of four repeats. The sequences of these primers were listed in the Table S2.

***In vitro* reporter gene assay.** Cells transfected with plasmids harboring luciferase reporter gene were lysed and harvested using the passive lysis buffer (Promega Corporation, Madison, WI, USA). The luciferase activity was analyzed using the reporter assay buffer (50mM Glycylglycin, 1M MgSO_4_, 10mg/ml of BSA, and 0.5M EDTA) mixed with 100mM ATP (Sigma-Aldrich, St. Louis, MO, USA), 1M dithiothreitol, and 50mM D-Luciferin (Xenogen, Challenger Dr, Alameda, CA, USA), and then measured using a multi-label counter (Perkin Elmer Wallac 1420 victor^2^, Waltham, MA, USA). The unit of all bioluminescent signals was count per second (CPS) and normalized to protein concentrations of cell lysates.

**Chromatin immunoprecipitation–polymerase chain reaction (ChIP-PCR) assay.** The ChIP assay protocols were followed by the manufacturer’s instruction of ChIP assay kit (Millipore, Billerica, MA, USA). Briefly, approximately 1x10^6^ cells were incubated in 1% formaldehyde at 37°C for 10 minutes followed by triple washes by cold PBS containing 1% cocktail protease inhibitor (Calbiochem, San Diego, CA, USA). The lysate was then sonicated to shear the chromatin to 200 to 1000 bps length fragments. The pre-cleared chromatin solution was incubated with specific anti-TEAD1 antibody (Cell Signaling Technology, Danvers, MA, USA), or anti-rabbit IgG1 antibody (Genetex Inc., Irvine, CA, USA) as negative control overnight. The immunocomplexes were captured with ssDNA/Protein A agarose beads. After extensive wash, the bound DNA fragments were eluted following reverse crosslink by heat treatment (at 65°C for 4hours) and proteinase K digestion (at 45°C for 1 hour) for further purification of DNA samples. The eluted genomic DNA fragments were analyzed by conventional PCR or qPCR (Lin et al., 2017) as mentioned above. The fold enrichment of TEAD1-promoter interaction was determined by the 2^–ΔΔCT^ formula. The following primer sets were used for PCR: TEAD1 binding segment F: 5’- GCAGACCACGAGGTGGGGGCCGCTG-3’ and R: 5’- CCGGGACCTGGACCAGAGGACCGCG-3’; Negative control F: 5’– AAAAGTAGAAAGGGACGAGTTCCCA-3’ and R: 5’- GCTGTCTCAGACACGTTTAGTTTTG -3’. The PCR products were run on 2% agarose gel.

**Animals.** Male Balb/cByJNarl mice were purchased from National Laboratory Animal Center (NLAC), Taipei, Taiwan. Mice were mainly used for excision of different tissues shown in this study (N=3). The animal studies and experiments were approved by the Institutional Animal Care and Use Committee (IACUC) in National Yang-Ming University, Taipei, Taiwan. The IACUC approval number is 1011101.

**Human Lung Tissues.** Human lung tissues were obtained from 50 patients at different ages in Mackey memorial hospital, Taipei, Taiwan. These donors were not lung cancers patients but other lung related diseases such as pulmonary emphysema. These samples were fixed with 4% paraformaldehyde in 4℃ with gentle shaking overnight. Tissues embedded in frozen tissue matrix (OCT^®^) were stored in -80℃. The samples were disconnected from donors’ private records. Use of human tissue sections for IHC staining was approved by the institutional review board of Mackay Memorial Hospital (16MMHIS073e).

**Statistical Analysis.** Each datum represented the mean ± S.D. of three independent experiments except those indicated in the legend. Statistical analysis between the control and experimental groups or between experimental groups with different conditions were determined using *t* test. It was assumed that the data distribution was normal. Analysis of variance (ANOVA) test was also used to analyse cell growth ratio after knockdown of cofilin-1 and change of telomere length. The p value < 0.05 was considered statistic significance.

Campenhout, C. V., Cabochette, P., Veillard, A. C., Laczik, M., Zelisko-Schmidt, A., Sabatel, C., . . . Kruys, V. (2019). Guidelines for optimized gene knockout using CRISPR/Cas9. *Biotechniques, 66*(6), 295-302. doi:10.2144/btn-2018-0187

Houghton, B. A., & Stidworthy, G. H. (1979). A growth history comparison of the human diploid cells WI-38 and IMR-90: proliferative capacity and cell sizing analysis. *In vitro, 15*(9), 697-702.

Hung, S. C., Chen, N. J., Hsieh, S. L., Li, H., Ma, H. L., & Lo, W. H. (2002). Isolation and characterization of size-sieved stem cells from human bone marrow. *Stem Cells, 20*(3), 249-258. doi:10.1634/stemcells.20-3-249

Lee, Y. J., Mazzatti, D. J., Yun, Z., & Keng, P. C. (2005). Inhibition of invasiveness of human lung cancer cell line H1299 by over-expression of cofilin. *Cell Biol Int, 29*(11), 877-883. doi:10.1016/j.cellbi.2005.07.005

Lin, Y. C., Chang, Y. T., Campbell, M., Lin, T. P., Pan, C. C., Lee, H. C., . . . Chang, P. C. (2017). MAOA-a novel decision maker of apoptosis and autophagy in hormone refractory neuroendocrine prostate cancer cells. *Sci Rep, 7*, 46338. doi:10.1038/srep46338

Minami, S., Ohtani-Fujita, N., Igata, E., Tamaki, T., & Sakai, T. (1997). Molecular cloning and characterization of the human p27Kip1 gene promoter. *FEBS Lett, 411*(1), 1-6.

O'Callaghan, N., Dhillon, V., Thomas, P., & Fenech, M. (2008). A quantitative real-time PCR method for absolute telomere length. *Biotechniques, 44*(6), 807-809. doi:10.2144/000112761

Tahtamouni, L. H., Shaw, A. E., Hasan, M. H., Yasin, S. R., & Bamburg, J. R. (2013). Non-overlapping activities of ADF and cofilin-1 during the migration of metastatic breast tumor cells. *BMC Cell Biol, 14*, 45. doi:10.1186/1471-2121-14-45

Tsai, C. H., Chiu, S. J., Liu, C. C., Sheu, T. J., Hsieh, C. H., Keng, P. C., & Lee, Y. J. (2009). Regulated expression of cofilin and the consequent regulation of p27(kip1) are essential for G(1) phase progression. *Cell Cycle, 8*(15), 2365-2374. doi:10.4161/cc.8.15.9072

Tsai, C. H., Lin, L. T., Wang, C. Y., Chiu, Y. W., Chou, Y. T., Chiu, S. J., . . . Lee, Y. J. (2015). Over-expression of cofilin-1 suppressed growth and invasion of cancer cells is associated with up-regulation of let-7 microRNA. *Biochim Biophys Acta, 1852*(5), 851-861. doi:10.1016/j.bbadis.2015.01.007

Yoo, Y., Ho, H. J., Wang, C., & Guan, J. L. (2010). Tyrosine phosphorylation of cofilin at Y68 by v-Src leads to its degradation through ubiquitin-proteasome pathway. *Oncogene, 29*(2), 263-272. doi:10.1038/onc.2009.319
